# The impact of tumor epithelial and microenvironmental heterogeneity on treatment responses in HER2^+^ breast cancer

**DOI:** 10.1172/jci.insight.147617

**Published:** 2021-06-08

**Authors:** Michalina Janiszewska, Shayna Stein, Otto Metzger Filho, Jennifer Eng, Natalie L. Kingston, Nicholas W. Harper, Inga H. Rye, Maša Alečković, Anne Trinh, Katherine C. Murphy, Elisabetta Marangoni, Simona Cristea, Benjamin Oakes, Eric P. Winer, Ian E. Krop, Hege G. Russnes, Paul T. Spellman, Elmar Bucher, Zhi Hu, Koei Chin, Joe W. Gray, Franziska Michor, Kornelia Polyak

**Affiliations:** 1 Department of Medical Oncology, Dana-Farber Cancer Institute, Boston, Massachusetts, USA.; 2 Department of Medicine, Brigham and Women’s Hospital, and Department of Medicine, Harvard Medical School, Boston, Massachusetts, USA.; 3 Department of Molecular Medicine, The Scripps Research Institute, Jupiter, Florida, USA.; 4 Department of Data Science, Dana-Farber Cancer Institute, Boston, Massachusetts, USA.; 5 Department of Biostatistics, Harvard T.H. Chan School of Public Health, Boston, Massachusetts, USA.; 6 OHSU Center for Spatial Systems Biomedicine, Department of Biomedical Engineering, School of Medicine, Oregon Health and Science University, Portland, Oregon, USA.; 7 OHSU Knight Cancer Institute, Oregon Health and Science University, Portland, Oregon, USA.; 8 Department of Pathology, Division of Laboratory Medicine, and Department of Cancer Genetics, Institute for Cancer Research, Division of Cancer Medicine, Oslo University Hospital, Oslo, Norway.; 9 Department of Translational Research, Institut Curie, Paris, France.; 10 Department of Stem Cell and Regenerative Biology, Harvard University, Cambridge, Massachusetts, USA.; 11 Department of Molecular and Medical Genetics, School of Medicine, Oregon Health and Science University, Portland, Oregon, USA.; 12 Center for Cancer Evolution, Dana-Farber Cancer Institute, Boston, Massachusetts, USA.; 13 The Broad Institute of MIT and Harvard, Cambridge, Massachusetts, USA.; 14 Ludwig Center at Harvard Medical School, Boston, Massachusetts, USA.

**Keywords:** Oncology, Breast cancer, Molecular pathology

## Abstract

Despite the availability of multiple human epidermal growth factor receptor 2–targeted (HER2-targeted) treatments, therapeutic resistance in HER2^+^ breast cancer remains a clinical challenge. Intratumor heterogeneity for HER2 and resistance-conferring mutations in the *PIK3CA* gene (encoding PI3K catalytic subunit α) have been investigated in response and resistance to HER2-targeting agents, while the role of divergent cellular phenotypes and tumor epithelial-stromal cell interactions is less well understood. Here, we assessed the effect of intratumor cellular genetic heterogeneity for *ERBB2* (encoding HER2) copy number and *PIK3CA* mutation on different types of neoadjuvant HER2-targeting therapies and clinical outcome in HER2^+^ breast cancer. We found that the frequency of cells lacking HER2 was a better predictor of response to HER2-targeted treatment than intratumor heterogeneity. We also compared the efficacy of different therapies in the same tumor using patient-derived xenograft models of heterogeneous HER2^+^ breast cancer and single-cell approaches. Stromal determinants were better predictors of response than tumor epithelial cells, and we identified alveolar epithelial and fibroblastic reticular cells as well as lymphatic vessel endothelial hyaluronan receptor 1–positive (Lyve1^+^) macrophages as putative drivers of therapeutic resistance. Our results demonstrate that both preexisting and acquired resistance to HER2-targeting agents involve multiple mechanisms including the tumor microenvironment. Furthermore, our data suggest that intratumor heterogeneity for HER2 should be incorporated into treatment design.

## Graphical abstract

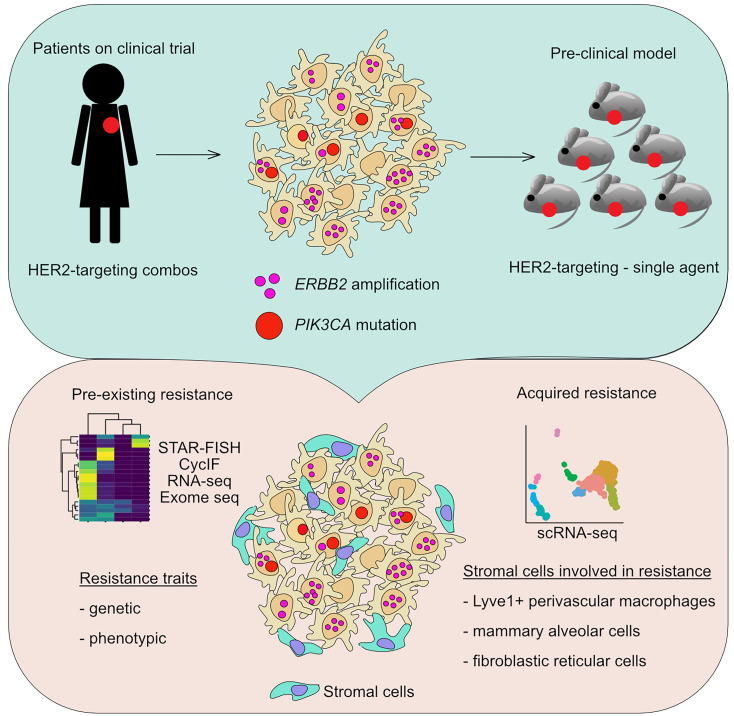

## Introduction

Amplification and overexpression of *ERBB2* encoding the human epidermal growth factor receptor 2 (HER2) distinguishes a subtype of breast cancers that accounts for approximately one-fifth of all invasive breast cancer cases (1). Inhibition of HER2 was one of the first examples for targeted cancer therapy based on the development and use of the anti-HER2 antibody trastuzumab (2). Over the past 2 decades, the combination of trastuzumab with chemotherapy became a standard of care for patients with HER2^+^ breast cancer. Even though this targeted approach substantially improves the disease-free and overall survival of patients with HER2^+^ breast cancer, virtually all patients with advanced HER2^+^ disease will eventually develop resistance and progressive disease. Thus, to further improve treatment efficacy, numerous other HER2-targeting agents have been developed and evaluated in the clinic, including various HER2 antibodies and small molecule inhibitors (SMIs) of the HER2 kinase (3). Trastuzumab and pertuzumab are 2 FDA-approved monoclonal antibodies that bind to the extracellular domain of HER2 and inhibit its activity while activating the antitumor immune response via antibody-dependent cellular cytotoxicity (3). HER2-targeting antibodies were also used to engineer antibody-drug conjugates (ADCs), such as trastuzumab emtansine (T-DM1) (4). Upon binding to HER2, T-DM1 is internalized into lysosomes, where it is degraded, releasing its microtubule inhibitor payload (DM1) directly into the HER2^+^ cancer cell. SMIs inhibiting the tyrosine kinase activity of HER2 receptor complexes, such as lapatinib, were also shown to have some activity in a subset of patients (3). Several potential mechanisms of resistance to HER2-targeted therapy have been identified from preclinical and clinical studies. These include genetic alterations, such as mutations in PI3K catalytic subunit α (*PIK3CA*) and *ERBB2* leading to constitutive activation of downstream signaling pathways (5). Upregulation of multiple other pathways, such as MET or SRC/FAK signaling, can also promote protumorigenic signaling during HER2 inhibition (6). However, despite accumulating knowledge in this area, the actual molecular changes driving resistance in human cancers have not been definitively demonstrated, and accurate predictions of the likelihood of resistance based on diagnostic biopsy profiles are not yet feasible.

A major obstacle to the effective treatment of HER2^+^ breast cancers is intratumor heterogeneity (ITH) for HER2 itself (7). The latest American Society of Clinical Oncology/College of American Pathologists guidelines support reporting of HER2 status as positive for tumors if at least 10% of the cancer cells stain positive for HER2 by immunohistochemistry (8). Thus, within each HER2^+^ tumor, there may be many cancer cells that lack HER2 and are genetically and functionally different from their HER2^+^ counterparts. We previously described a novel method, specific-to-allele PCR-FISH (STAR-FISH), to assess cellular genetic heterogeneity for *ERBB2* copy number and the *PIK3C*A H1047R hotspot mutation in a small cohort of HER2^+^ tumors subjected to neoadjuvant chemotherapy (9). Using this approach, we showed that patients with a significant increase in spatial cellular genetic heterogeneity after neoadjuvant treatment had shorter recurrence-free survival compared with patients with no change. Furthermore, 2 patients who received a combination of neoadjuvant chemotherapy and trastuzumab showed a decrease in spatial cellular genetic heterogeneity, yet still had poor outcome, implying the presence of preexisting subpopulations resistant to HER2-targeted therapies. However, how ITH impacts and is impacted by different types of HER2-targeted therapies remain unclear. In this study, we explored the effects of intratumor cellular heterogeneity for *ERBB2* copy number and *PIK3C*A^H1047R^ on the response to different types of HER2-targeting therapeutic strategies and changes in this heterogeneity during treatment in human breast tumors and patient-derived xenografts (PDXs).

## Results

### Cellular genetic heterogeneity and response to neoadjuvant HER2-targeting therapies.

**Figure 1:**
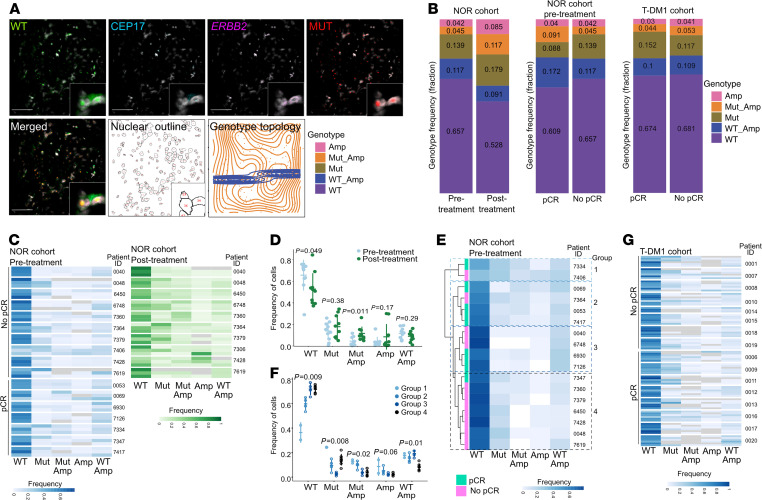
Cellular genetic heterogeneity in neoadjuvant HER2-targeted treatment patient cohorts. (**A**) Representative image of STAR-FISH analysis. Nuclear outline image and topological map of the sample are shown. Scale bars: 50 μm. WT, *PIK3CA* wild-type STAR-FISH signal; CEP17, chromosome 17 centromeric probe; *ERBB2*, *ERBB2*-specific FISH probe; MUT, PIK3CA mutant; Amp, amplification of *ERBB2*. (**B**) Summary of frequencies of cells with distinct genotypes in Norwegian (NOR) and T-DM1 cohorts. pCR, pathological complete response; No pCR, no pathological complete response. (**C**) Frequencies of cells with distinct genotypes in each analyzed sample from NOR cohort. Each row corresponds to a single image analyzed (*n* = 3 per case). Gray represents a frequency of 0. Images are grouped according to the patient ID, and patient IDs are grouped according to response (left). For nonresponders, frequency of genotypes after treatment is also shown (right). (**D**) Average genotype frequency in pre- versus posttreatment samples from NOR cohort. *P* values from a Wilcoxon test comparing the change in frequency pre- and posttreatment to 0. (**E**) Unsupervised clustering of frequencies of cells with distinct genotypes per patient in pretreatment samples from Norwegian cohort. Samples are colored according to response. (**F**) Differences in genotype frequencies between groups identified in (**E**). *P* values from Kruskal-Wallis test. (**G**) Frequencies of cells with distinct genotypes in each analyzed sample from T-DM1 cohort. Images are grouped according to the patient ID, and patient IDs are grouped according to response.

To assess the associations between cellular heterogeneity for *ERBB2* copy number and mutant *PIK3CA*^H1047R^ as well as response to treatment and changes in heterogeneity due to therapy, we performed STAR-FISH on cases from 2 uniformly treated cohorts of patients with HER2^+^ breast cancer. One cohort is from Norway (NOR cohort) and consists of 30 patients treated with neoadjuvant chemotherapy and trastuzumab, 10 of which have both pre- and posttreatment samples; the other cohort (T-DM1 cohort) contains 16 patients treated with neoadjuvant T-DM1 and pertuzumab (7) (Supplemental Figure 1, A and B, and Supplemental Table 1; supplemental material available online with this article; https://doi.org/10.1172/jci.insight.147617DS1). To assess spatial heterogeneity, we analyzed both pre- and posttreatment biopsies in the NOR cohort, whereas in the T-DM1 cohort only pretreatment samples were analyzed, with 2 spatially separate biopsies per case. We utilized STAR-FISH, a combination of mutation-specific in situ PCR and FISH that allows for detection of point mutation and copy number alterations at the single-cell level in an intact archival sample (9) (Figure 1A). The signals from each fluorescent channel were quantified in individual nuclei, and cells were assigned to 1 of 5 genotypes reflecting *ERBB2* amplification and *PIK3C*A^H1047R^ mutation status (Amp, Mut-Amp, Mut, WT-Amp, and WT; see Methods for details). The genotypes were then projected as genotype topology maps (Figure 1A). The number of cells quantified in each biopsy varied; thus, we compared relative genotype frequencies across samples and patients rather than absolute cell numbers.

First, we investigated whether the frequency of cells with *ERBB2* amplification (Amp) and *PIK3CA*^H1047R^ (Mut) changes during neoadjuvant treatment. We observed a significant enrichment for cells with the Mut-Amp genotype in the posttreatment as compared with the pretreatment samples in the NOR cohort (Figure 1B), both in the entire cohort (*P* = 0.01, mean frequency change 0.0723) and when individual cases were analyzed separately (Figure 1, C and D; Supplemental Figure 2, A and B; and Supplemental Table 2). The fraction of Amp cells did not change significantly (*P* = 0.173, mean frequency change 0.0291), while the frequency of WT cells was significantly lower after treatment (*P* = 0.0497, mean frequency change –0.129). The spatial distribution of cells with different genotypes, based on recorded coordinates for each individual nucleus analyzed and measured by k-means clustering (see Methods), was not significantly altered during treatment (Supplemental Figure 2C).

We then investigated whether the frequency of cells with a certain genotype in pretreatment samples was associated with response to neoadjuvant therapy defined at the time of surgical excision as complete pathologic response (pCR) or no-pCR. We performed hierarchical clustering of patients using pretreatment samples only. Due to the variability between different regions within the same sample (Figure 1C and Supplemental Figure 2A), we used the overall genotype frequency for each patient, rather than using each sampled region individually. We identified 4 major clusters (groups), with group 4 enriched in no-PCR cases (Figure 1E). While the genotype frequencies differed significantly between the 4 groups, we detected no clear association between group 4 and the other groups (Figure 1, E and F), suggesting there was no discernable difference between pCR and no-pCR groups based on pretreatment biopsies. Both groups of samples had a similar spatial distribution of cells with different genotypes, except for WT-Amp cells, which were more dispersed in tumors with pCR (Supplemental Figure 2D). Thus, although we did observe an enrichment of Mut-Amp cells in the residual tumors, higher frequency of these cells in pretreatment samples did not significantly impact the response to neoadjuvant therapy.

We have also explored potential associations between genotype heterogeneity and clinico-pathological information. Hormone receptor status of all tested samples did not correlate with changes in frequency of cells with distinct genotypes, except for cases with high HER2 expression having higher frequency of Mut-Amp cells, compared with cases with medium HER2 expression (*P* = 0.02; Supplemental Figure 2E). There was also no association between the overall heterogeneity of the pretreatment tumor samples from both responders and nonresponders and the presence of distant metastases (*P* = 0.27; Supplemental Figure 2F).

Next, we investigated whether the overall heterogeneity of the pretreatment tumor samples or a change in the extent of tumor heterogeneity pre- and posttreatment reflects a risk for breast cancer–specific death. While in many cases, there was a significant change in tumor heterogeneity in pre- versus posttreatment samples, there was no significant association between the change in tumor heterogeneity and survival, a finding potentially caused by small sample size (Supplemental Figure 3, A and B). There was also no significant association between the overall heterogeneity of the pretreatment tumor samples from both pCR and no-pCR cases and long-term overall survival, although patients with higher heterogeneity pretreatment appeared to have better survival (Supplemental Figure 3C). This observation was unexpected because higher diversity is generally associated with poor outcomes. Upon further investigation, we found that higher diversity tumors had significantly more cells with *ERBB2* amplification (*P* = 0.0079) and significantly fewer cells without the *ERBB2* amplification or *PIK3CA* mutation (*P* = 0.0003; Supplemental Figure 3D). This suggests that the lower diversity tumors in this patient cohort consisted primarily of HER2^–^ cells that did not respond to HER2-targeted therapy. The higher diversity tumors, on the other hand, had larger populations of *ERBB2*-amplified cells and thus responded better to HER2-targeted therapy. Moreover, we found that patients with a higher level of *ERBB2* amplification had better survival outcomes, although this difference was not significant likely because of the small sample size (*P* = 0.8; Supplemental Figure 3E).

Similarly, in the T-DM1 cohort, the frequency of cells with distinct genotypes was not significantly different between patients who achieved pCR and those with no-pCR (*P* = 0.65 for Amp, *P* = 0.51 for Mut, *P* = 0.38 for Mut-Amp, *P* = 0.1 for WT, *P* = 0.57 for WT-Amp; Figure 1G and Supplemental Figure 4, A and B). Similar to the NOR cohort, untreated samples from patients with pCR and no-PCR in the T-DM1 cohort had similar spatial distributions of cells with distinct genotypes, and the overall pretreatment heterogeneity did not stratify patients by response to neoadjuvant therapy (*P* = 0.56; Supplemental Figure 4, C and D). We also did not find significant associations between pretreatment genotype frequency and hormone receptor and HER2 status (Supplemental Figure 4E). Since this T-DM1 trial is ongoing, we were not able to analyze associations with long-term outcome.

### Cellular phenotypic heterogeneity and response to neoadjuvant HER2-targeting therapies.

**Figure 2:**
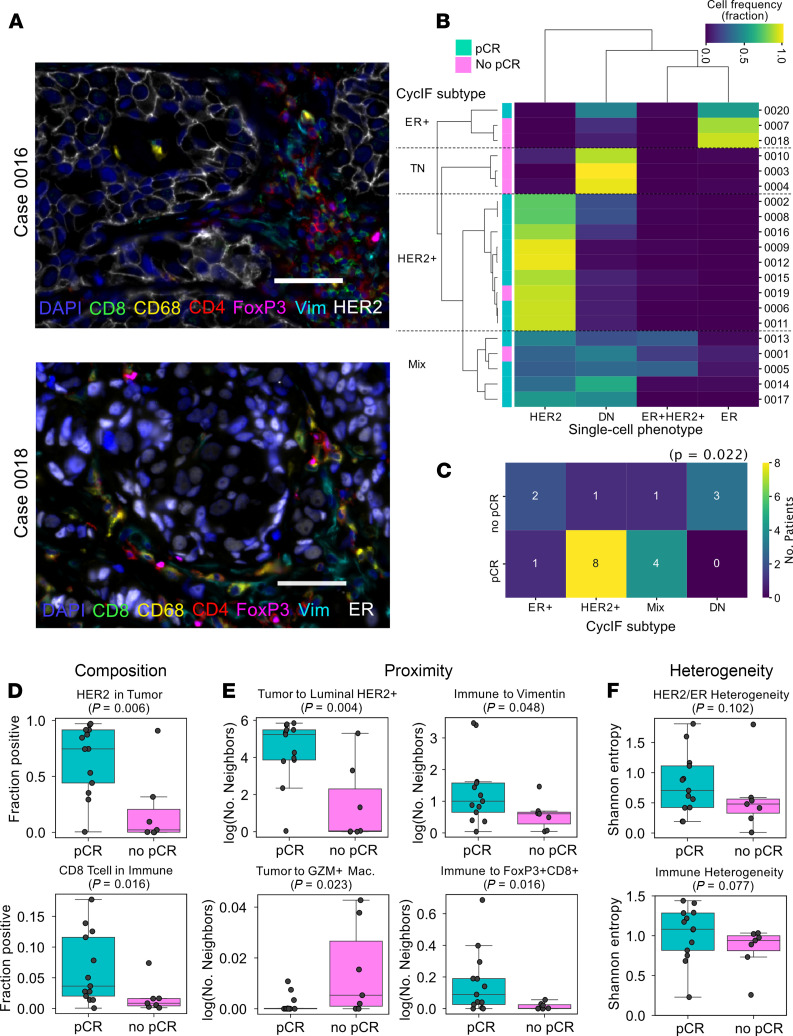
Phenotypic single-cell diversity measured by CycIF. (**A**) Representative CycIF staining in HER2^+^ case 0016 (top) and ER^+^ case 0018 (bottom). Vim, Vimentin. Scale bars: 50 μm. (**B**) Hierarchical clustering of samples based on HER2 and ER positivity in tumor cells. pCR, pathological complete response; DN, double-negative (ER^–^HER2^–^). (**C**) Association between CycIF subtype identified in **B** and pCR. HER2^+^ and mixed tumors had significantly better response than DN or ER^+^ (χ^2^, *P* = 0.022). (**D**–**F**) Associations between tumor and immune measurements of composition (**D**), proximity (**E**), and heterogeneity (**F**) and pCR. Wilcoxon’s rank-sum test *P* values are shown. GZM, granzyme B.

To test whether phenotypic heterogeneity could be a better predictor of response to neoadjuvant therapy than metrics based on genetic features, we performed cyclic immunofluorescence (CycIF) for 22 protein markers on consecutive slides of pretreatment samples from the T-DM1 cohort (Figure 2A). This allowed us to classify individual cells within each tissue sample as tumor, stroma, or immune subtype based on marker combinations (Supplemental Table 3). Tumor cells were classified according to their expression of HER2 and ER into 4 categories (Figure 2, B and C). The frequency of these cell types in each case correlated with response to neoadjuvant therapy, with pCR cases having higher frequency of HER2^+^ cells and no-pCR cases having higher proportion of ER^–^HER2^–^ cells (χ^2^ test *P* = 0.022, Figure 2, B–D). Moreover, pCR was also associated with increased fraction of CD8^+^ T cells among tumor-infiltrating immune cells (*P* = 0.016, Figure 2D). Next, we asked if the proximity between distinct cell types could be predictive of response to neoadjuvant therapy (Figure 2E). We found that tumors with pCR were characterized by more frequent contacts between tumor cells and tumor cells of luminal HER2^+^ subtype (*P* = 0.004). Tumors with no-PCR had higher frequency of tumor cells neighboring GZM^+^ macrophages (*P* = 0.023). The immune cells in tumors with no-pCR also had fewer contacts with vimentin^+^ stromal cells (nontumor, nonimmune, nonendothelial cell gate, *P* = 0.048), potentially representing a mesenchymal and more migratory phenotype, than those in pCR cases. Moreover, immune cells in no-pCR cases had significantly (*P* = 0.016) fewer neighboring FoxP3^+^CD8^+^ regulatory T cells compared with tumor tissues with pCR. Thus, our results suggest that resistance to combined T-DM1 and pertuzumab treatment might be associated with impaired immune targeting and killing of tumor cells.

Using the single-cell CycIF quantitative data, we tested whether ITH for expression of HER2 and ER protein or the diversity of immune cells within a tumor could stratify the samples according to response to neoadjuvant treatment. Neither of the heterogeneity metrics showed a significant association with pCR (Figure 2F). There was also no significant correlation between HER2 expression by CycIF and *ERBB2* amplification measured by STAR-FISH (Supplemental Figure 5A), and we did not find significant trends in CycIF data that were associated with the frequency of *PIK3CA*-mutant cells (Supplemental Figure 5, B and C). Since the T-DM1 trial is ongoing, it remains to be seen if cellular ITH for *ERBB2* and *PIK3CA*^H1047R^ or the phenotypic and immune microenvironment diversity is associated with long-term clinical outcome.

### Variable responses to single-agent HER2-targeted therapies in PDX models.

**Figure 3:**
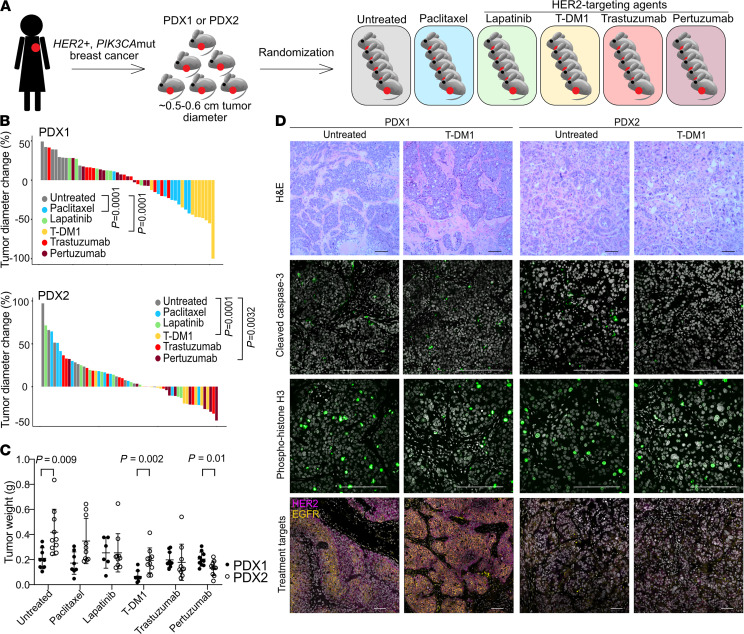
Single-agent *HER2*-targeted treatment effects on 2 HER2^+^ *PIK3CA*^mut^ PDXs. (**A**) Schematic of experimental design. (**B**) Treatment response for PDX1 and PDX2. Waterfall plots show percentage change in diameter for each tumor; *n* = 10 tumors and 5 animals per treatment group. (**C**) Tumor weight at the experimental endpoint. *P* values in **B** and **C** indicate statistical significance based on unpaired 2-tailed Student’s *t* tests. (**D**) Representative images of the histology (hematoxylin and eosin staining; upper panels) and immunofluorescence staining for apoptosis marker (cleaved caspase-3), cell proliferation marker (phospho-histone H3), and treatment targets (HER2 and phospho-EGFR proteins). Scale bars: 100 μm. Staining was repeated twice with similar results.

Our STAR-FISH data showed that cells with *PIK3CA*^H1047R^ are often present in untreated HER2^+^ breast tumors as minor subpopulations that increase in frequency after neoadjuvant chemotherapy with or without a HER2-targeted agent (Figure 1B), implying that mutant *PIK3CA* might play a role in therapeutic resistance (9). In order to better understand the effects of mutant *PIK3CA* on response and resistance to different types of therapies, the same tumor should be treated with different agents, which is not possible in the clinic. Thus, we tested the response of 2 different HER2^+^ *PIK3CA*-mutant breast cancer PDXs to paclitaxel and 4 different *HER2*-targeting agents (lapatinib, T-DM1, trastuzumab, and pertuzumab). PDX1 was derived from untreated HER2^+^ breast cancer, carrying E365K mutation in C2 PI3K-type domain of *PIK3CA* gene, while PDX2, with *PIK3CA* H1047R hotspot mutation in kinase domain, was derived from a tumor pretreated with several rounds of trastuzumab with vinorelbine, another tubulin-binding chemotherapeutic drug (see Methods for details). Both patients received trastuzumab after tumor resection; however, their disease course differed dramatically, resulting in stable remission (PDX1) versus metastatic disease and death (PDX2). To generate a treatment cohort, we performed mammary fat pad injections in NOD.Cg-*Prkdc^scid^ Il2rg^tm1Sug^*/JicTac (NOG) mice (*n* = 30 for each PDX, 2 injections, both inguinal mammary fat pads in each animal). Once all tumors reached over 0.5 cm diameter, the animals were randomized to 6 treatment groups (*n* = 5 animals per group) and treated for 3 weeks (Figure 3A and Supplemental Figure 6A). None of the treatments induced a complete tumor regression of either PDX model in this time frame. The most significant decrease in tumor volume was observed with T-DM1 treatment in both PDX models (*P* = 0.0001, mean fold change 2.3 for PDX1 and 1.3 for PDX2; Figure 3, B and C; Supplemental Figure 6, B and C; and Supplemental Table 4). The most pronounced difference between the 2 PDXs was their response to paclitaxel where only PDX1 showed a decrease in size during treatment (*P* = 0.0001). PDX1 was also significantly more sensitive to T-DM1 (Figure 3, B and C). T-DM1 is a conjugate of trastuzumab with emtansine, which exerts its cytotoxic function by binding to tubulin (10). Paclitaxel is also a tubulin-binding agent, and thus the higher sensitivity of PDX1 to both T-DM1 and paclitaxel might be due to the antitubulin effects of these agents. PDX2 was significantly more sensitive to pertuzumab than PDX1 (*P* = 0.0032, mean fold change 1.2 for PDX1 and 1.5 for PDX2; Figure 3, B and C), and both tumor models were refractory to trastuzumab and lapatinib treatment (*P* = 0.11 and 0.24 for PDX1, *P* = 0.1 and 0.35 for PDX2, respectively).

To assess treatment-induced changes and explore mechanisms of resistance, we analyzed the cellular and molecular profiles of the residual tumors. The histology of T-DM1- and paclitaxel-treated samples showed some differences in the PDX1 model, with T-DM1 resulting in more stroma admixed within the tumor (Figure 3D and Supplemental Figure 7A), but none of the other treatments affected tumor cellularity. Cell death and proliferation rates measured by cleaved caspase-3 and phospho–histone H3 immunofluorescence were essentially the same in all treatment groups as in untreated controls (Figure 3D; Supplemental Figure 7, A–C; and Supplemental Table 5). These results suggest that after 3 weeks of treatment, all residual tumor cells might be therapy-resistant and that PDX1 and PDX2 differ in their initial responses to certain drugs. We reasoned that the residual tumors can be classified into 2 groups with distinct resistance mechanisms: (a) preexisting resistance to treatments that had no effect on tumor growth (groups treated with lapatinib, trastuzumab, and pertuzumab for PDX1 and paclitaxel, lapatinib and trastuzumab for PDX2) and (b) adaptive resistance related to regression in tumor size because of elimination of treatment-sensitive cells. Given the distinct disease history of our PDXs, our observations suggest that strong selective and adaptive mechanisms specific for each patient’s tumor may determine long-term outcomes.

### Genetic mechanisms of resistance to HER2-targeted therapies.

One mechanism by which cancer cells can evade targeted therapy is the lack or loss of the target in tumor cells (11). HER2^+^ tumors often display cellular or spatial heterogeneity for *ERBB2* gene amplification and overexpression (12). Thus, HER2-targeting therapies may select for cancer cells with fewer *ERBB2* copies or lower levels of HER2 protein expression. Indeed, our CycIF data showed that patients with higher fraction of HER2-negative tumor cells prior to treatment are less likely to achieve pCR (Figure 2, B and D). To explore if this observation could explain the relative treatment resistance of our PDX models, we performed FISH to assess *ERBB2* copy number and immunofluorescence for HER2, phospho-EGFR, and ER, which have been associated with treatment responses in patients (12). We did not observe any noticeable differences in the expression of these proteins due to treatment in either PDX model, and both PDXs retained high levels of *ERBB2* amplification in all treatment groups (Figure 3D and Supplemental Figure 7, A–E). These results suggest that treatment resistance in these PDX models cannot be explained by the lack of drug target (i.e., HER2) expression.

Next, we performed exome sequencing (exome-Seq) of untreated and treated PDX1 and PDX2 samples to identify preexisting or acquired mutations that could explain the differences in response to various treatments. Both PDXs were *PIK3CA* mutant, and exome-Seq confirmed the presence of the *PIK3CA* E365K mutation in PDX1 and of the H1047R mutation in PDX2 (Supplemental Figure 8A). The allelic frequency of these *PIK3CA* mutations was close to 1 in all tumors tested, except in PDX1 treated with T-DM1, where a decrease to 0.75 was observed in both sequenced samples. Thus, changes in the frequency of *PIK3CA*-mutant cells could not explain the observed differences in response to HER2-targeted treatments.

To investigate whether the residual tumors acquired new mutations after treatment, we compared single nucleotide variants (SNVs) of treated tumors to the untreated controls. We set out to identify mutations that would be present in both replicates (2 independent tumors) within a treatment group compared with the untreated samples. In PDX1, we identified 10 nonsynonymous mutations that were present in both sequenced tumors from each treatment group (Supplemental Table 6). Only 3 out of these 10 mutations were pathogenic according to the Catalogue Of Somatic Mutations In Cancer (COSMIC) database, namely ankyrin repeat domain 17 (*ANKRD17*) p.S785N, trinucleotide repeat containing adaptor 6A (*TNRC6A*) p.M1320T, and ubiquitin specific peptidase 34 (*USP34*) p.F2756L (Supplemental Figure 8B). Except for the *TNRC6A* mutation, no other mutations were common between PDX1 and PDX2. TNRC6A is a component of the miRNA processing machinery also involved in RNA splicing and is frequently mutated in colorectal and gastric tumors with microsatellite instability (13). *TNRC6A* mutations were not present in all samples; however, almost every treatment group had a single tumor sample with this alteration, suggesting it may represent a more general resistance mechanism. ANKRD17 is a mask protein that regulates the nuclear import of the YAP proteins (14) and is also a substrate of CDK2, which plays a role in DNA replication during G/S cell cycle transition (15). USP34 was shown to inhibit stemness and epithelial-to-mesenchymal transition (EMT) in breast epithelial cells (16). In PDX1, the variant allele frequencies of the *ANKRD17* and *USP34* mutations were higher in T-DM1–treated samples compared with all other treatments and untreated tumors, while in PDX2 the same mutations were found in only 1 of 2 sequenced samples and had lower frequencies compared with PDX1 (Supplemental Figure 8B).

We did not detect any new nonsynonymous mutations in T-DM1–treated PDX2 samples, suggesting that either epigenetic or microenvironmental factors may be responsible for acquisition of resistance in this model or that the tumor already acquired resistance in the patient. Residual tumors after pertuzumab treatment harbored only 1 new pathogenic mutation, AT-rich interaction domain 4B (*ARID4B*) p.V600A. ARID4B is a subunit of the histone deacetylase–dependent SIN3 repressor complex and promotes mammary tumorigenesis and metastasis (17). This observation again points to epigenetic remodeling as a potential mechanism for acquired resistance in PDX2. Interestingly, trastuzumab- and lapatinib-treated PDX2 tumors that did not show differences in tumor growth did acquire novel mutations, many of which have not previously been reported in the COSMIC database (Supplemental Table 7). These findings suggest that different HER2-targeting therapies exert distinct evolutionary pressures on the tumors.

### Intrinsic resistance to HER2-targeted therapy is linked to activation of signaling pathways.

**Figure 4:**
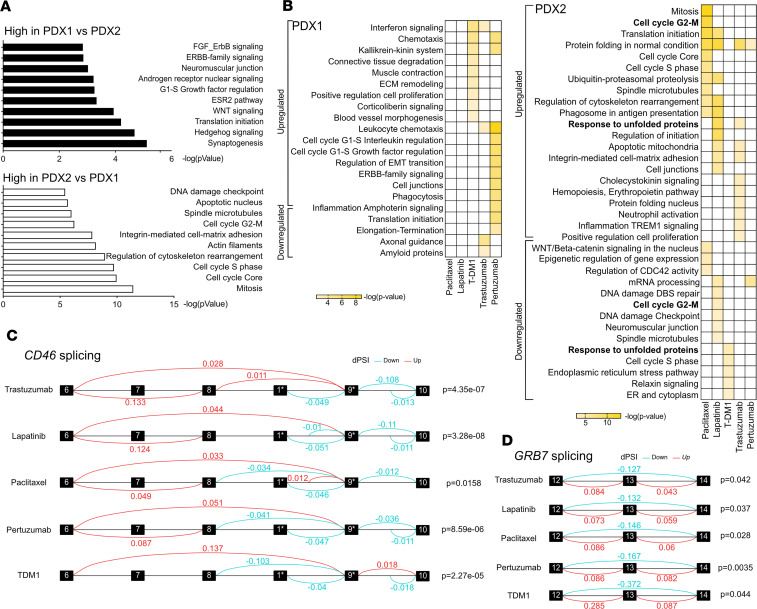
Gene expression profiles of HER2^+^ PDX models. (**A**) MetaCore Gene Ontology (GO) Processes overrepresented in expression profiles of untreated PDX1 compared with untreated PDX2 (top) and untreated PDX2 compared with untreated PDX1 (bottom). *n* = 3 independent tumors per group. The *x* axis corresponds to –log *P* value of the significance of enrichment, calculated using the MetaCore enrichment analysis. (**B**) MetaCore GO Processes upregulated and downregulated upon treatment compared with untreated controls. *n* = 3 independent tumors per group. The color scale corresponds to –log *P* value of the significance of enrichment, calculated using the MetaCore enrichment analysis. (**C** and **D**) Alternative splicing analysis of CD46 (**C**) and GRB7 (**D**) in PDX2. Boxes represent the exons between which significant alternative splicing events were detected. Red lines, splicing occurred more frequently than in untreated samples; blue lines, splicing occurred less frequently than in untreated samples. The dPSI (change in percentage spliced in) is indicated above each line. *n* = 3 independent tumors per group. *P* values from the LeafCutter (52) algorithm are shown. *Exons 6–10 correspond to the exons in the canonical CD46 transcript. Exon 1 is the first exon in transcript ENST00000636114.1 and is not included in the canonical CD46 transcript. Exon 9 in the canonical transcript corresponds to the second exon in ENST00000636114.1.

Since the differential mutational patterns and acquisition of or selection for new mutations did not fully explain the observed treatment resistance, we next performed transcriptional profiling of the PDX tumors to address the intrinsic resistance to paclitaxel and HER2-targeting agents in our 2 PDX models. Genes highly expressed in untreated PDX1 compared with untreated PDX2 were significantly enriched in synaptogenesis, including neuroligin, neuregulin 3, and synaptotagmin genes; Hedgehog and WNT signaling, characterized by overexpression of GLI-1, Frizzled, Hedgehog, TCF/LEF, and WNT7B; as well as the ESR2 pathway and androgen receptor and ERBB-family signaling (Figure 4A and Supplemental Table 7). These pathways are known to be involved in resistance to HER2 signaling inhibition (6). Genes highly expressed in PDX2 compared with PDX1 were enriched in cell cycle, cytoskeleton, and DNA damage checkpoint-related functions, including tubulin, myosin II, Chk2, Cyclin B, and Aurora A.

Next, we compared the gene expression profiles of tumors from all treatment groups with corresponding untreated tumors separately for PDX1 and PDX2. In PDX1 the expression patterns of every treatment were enriched in different pathways, implying distinct mechanisms of resistance (Figure 4B and Supplemental Table 7). Only leukocyte chemotaxis, interferon signaling, and kallikrein-kinin system were shared in 2 treatment groups. High expression of kallikreins has previously been linked to tamoxifen and paclitaxel resistance in breast and ovarian cancer, respectively (18, 19). Leukocyte chemoattractants, such as CCL5/RANTES and CXCL16, may contribute to breast cancer progression and drug resistance by recruiting monocytes and macrophages (20–23). In PDX1 tumors treated with paclitaxel, where tumor growth was significantly affected, the number of differentially expressed genes was too low for pathway analysis. This finding suggests that the residual tumor represents the resistant fraction that rebounded to the original tumor state. Alternatively, it is also possible that the bulk sequencing does not fully capture more fine-grained differences within the residual tumors. We observed clearer differences in gene expression of PDX2 tumors where protein folding and cell cycle–related pathways were enriched in multiple treatment groups (Figure 4B, right; and Supplemental Table 7). Increased expression of the components of the protein folding machinery, especially chaperones such as HSP90, may promote the evolution of new heritable traits and have been previously implicated in emergence of resistance to targeted therapy in ER^+^ breast cancer (24). Thus, the overall upregulation of genes involved in protein folding and genes responsible for cell cycle progression in several treatment groups of PDX2 tumors points to the universal nature of these resistance mechanisms.

In our analysis of genetic variants that could contribute to drug resistance, we found a mutation in a splicing factor, TNRC6A, present in at least 1 tumor from all treatment groups but not in the untreated controls. Therefore, we performed an alternative splicing analysis of our bulk RNA sequencing data to assess which phenotypic traits could be attributed to changes in spliced isoforms. In PDX1, we found a fusion of *GJC3-AZGP1* genes and *PPP1R1B-STARD3* genes present only in T-DM1–treated samples but no other splicing events (Supplemental Table 8). Treated PDX2 tumors had multiple splice isoform alterations (Supplemental Table 8). Two genes, *CD46* and growth factor receptor bound protein 7 (*GRB7*), were differentially spliced in all PDX2 residual tumors compared with untreated controls (Figure 4, C and D). Alternative splicing of CD46, a transmembrane glycoprotein involved in innate and adaptive immune response, has been shown to produce various isoforms, with different O-glycosylation and variable cytoplasmic tails (25). Exclusion of exons 7–8 and exon 13 of CD46 was shown to be nonrandom in activated and memory/effector T cells (26), and it may play a role in immune evasion. GRB7 is an adapter protein involved in HER2 signaling, and its splice variants have been implicated in ovarian cancer progression (27). These findings suggest that altered splicing may affect immune response regulation as well as boost HER2 downstream signaling to promote drug resistance (28).

### Cancer cell subpopulations associated with acquired resistance.

Since bulk RNA sequencing may obscure the presence of minor preexisting resistant clones and does not allow for the accurate separation of tumor epithelial and stromal cells, we performed single-cell RNA sequencing (scRNA-Seq). Two tumors were profiled from each treatment group, with an average of 4521 cells per sample (Supplemental Table 9). Based on our cluster tree analysis, we used the cell clusters identified using a resolution of 0.3 (Supplemental Figure 9). Clustering of cells of human origin from each untreated tumor revealed the presence of 5 (PDX1) or 6 (PDX2) distinct clusters (Supplemental Figure 10 and Supplemental Figure 11, A and B). To identify the changes exerted by the different treatments on the distinct cancer cell subpopulations, we analyzed the frequencies of each cell cluster in each treatment group using χ^2^ tests. In PDX1, T-DM1 treatment, which reduced tumor growth, resulted in a significant decrease in the number of cluster 2 cells, characterized by high VEGFA expression (*P* = 3.69 × 10^8^; Supplemental Figure 10C and Supplemental Table 10). In PDX2, the main changes associated with treatments decreasing tumor growth rates were a higher frequency of cells in cluster 1 in T-DM1–treated tumors (*P* = 3.1 × 10^5^) and cluster 1 and cluster 4 had decreased cellularity in tumors after pertuzumab treatment (*P* = 0.0011 for cluster 1, *P* = 2.61 × 10^7^ for cluster 4; Supplemental Figure 10D and Supplemental Table 10).

**Figure 5:**
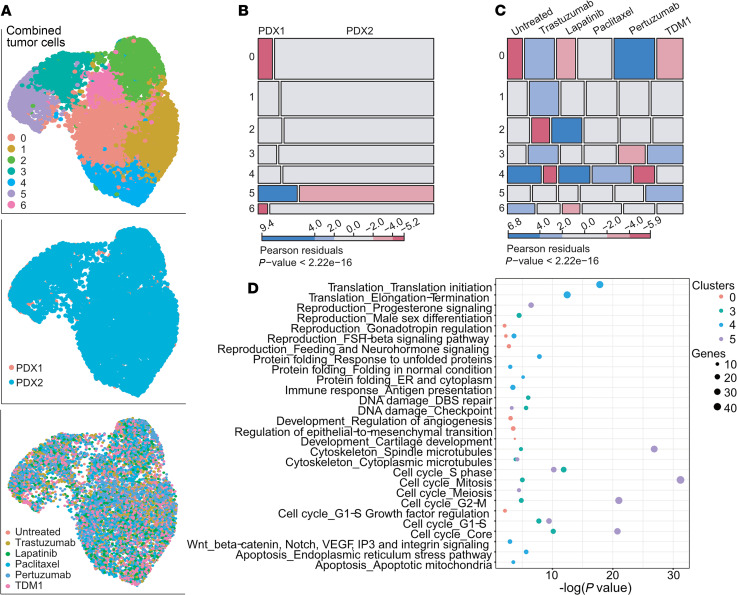
scRNA-Seq analysis of 2 PDXs identifies clusters associated with differential drug response. (**A**) Combined analysis of tumor cells from *n* = 2 samples per each PDX and each condition (total 24 samples, average cell number per sample = 2355). UMAP plots colored by cluster (top panel), PDX (middle panel), and treatment (bottom panel). (**B**) Cell distribution among clusters based on PDX from which they were derived. Red color, lower than expected frequency; blue, higher than expected. *P* value of χ^2^ test is shown. (**C**) Cell distribution in different clusters based on treatment. Red color indicates lower than expected frequency; blue, higher than expected. *P* value of χ^2^ test is shown. (**D**) MetaCore Pathway enrichment analysis for genes enriched in clusters differentially affected by distinct treatments (clusters 0, 3, 4, and 5). Circle size corresponds to number of genes found per pathway.

In search of common mechanisms of acquired resistance in the 2 PDX models, we performed clustering followed by Uniform Manifold Approximation and Projection (UMAP) dimensionality reduction of the combined data from both PDXs (Figure 5A and Supplemental Table 10). The contribution of cells from the 2 PDXs to each cluster was only significantly different for cluster 5 (*P* = 3.15 × 10^26^), as this cluster mostly contained cells from PDX1 (Figure 5B). The distribution of cells in clusters 0, 3, 4, and 5 was significantly influenced by treatment (Figure 5C). T-DM1 treatment, which affected the growth of both PDXs, resulted in a higher frequency of cells in cluster 3 (*P* = 0.004) and cluster 5 (*P* = 0.0003) compared with other treatment groups. Cluster 3 cells were also enriched in trastuzumab-treated samples (*P* = 0.004), yet pertuzumab-treated samples had fewer cells in this cluster (*P* = 0.014) compared with other treatment groups. Two HER2 antibody–based treatments, trastuzumab and pertuzumab, increased the frequency of cells in cluster 0 (*P*_trastuzumab_ = 1.08 × 10^5^, *P*_pertuzumab_ = 1.16 × 10^15^) and decreased the frequency of cells in cluster 4 (*P*_trastuzumab_ = 1.62 × 10^11^, *P*_pertuzumab_ = 1.88 × 10^7^) compared with untreated control.

To address the functional differences among clusters with differential treatment response, we performed process network analysis of genes most abundantly expressed in these clusters. We found that trastuzumab and pertuzumab treatment resistance was linked to survival of subpopulations of cancer cells able to regulate angiogenesis and EMT (cluster 0), while leading to elimination of cells with active translation, protein folding, and ER stress response linked to apoptosis (cluster 4; Figure 5D and Supplemental Table 10). The subpopulation of cells characterized by expression of DNA damage repair genes (cluster 3) was differentially affected by pertuzumab versus T-DM1 and trastuzumab, suggesting that DNA repair might be important for T-DM1 and trastuzumab resistance. Cluster 5 cells, enriched only in samples treated with T-DM1, had a strong cell cycle signature. Even though our immunofluorescence staining for phospho–histone H3 did not show overall changes in G2/M cells (Figure 3D and Supplemental Figure 6C), the scRNA-Seq data suggest an overrepresentation of cycling cells in T-DM1–resistant residual tumors.

Cell cycle signatures can be a major confounding factor in cluster identification in single-cell expression profiling experiments. Therefore, we investigated the relationship between the cell cycle and clustering and treatment effects. Cells in different phases of the cell cycle were indeed separated in the UMAP space. However, the only treatment-associated change was a decrease in S phase cell numbers after pertuzumab treatment (*P* = 0.012), when both PDXs were analyzed together (Supplemental Figure 11, A–C). Moreover, regressing out the cell cycle genes did not alter the clustering, suggesting that the biological processes found in our pathway analyses drive the cell cluster identity regardless of cell cycle involvement (Supplemental Figure 12). Overall, our scRNA-Seq profiling of the cancer cells from residual tumors identified several subpopulations of cells differentially affected by the treatments. Yet, none of the treatments resulted in the appearance of a new population or eradication of a specific cellular subtype after 3 weeks of treatment, suggesting that the acquisition of resistance is a complex process where a balance between different subpopulations might influence outcome.

### Stroma involvement in HER2-targeted treatment resistance.

**Figure 6:**
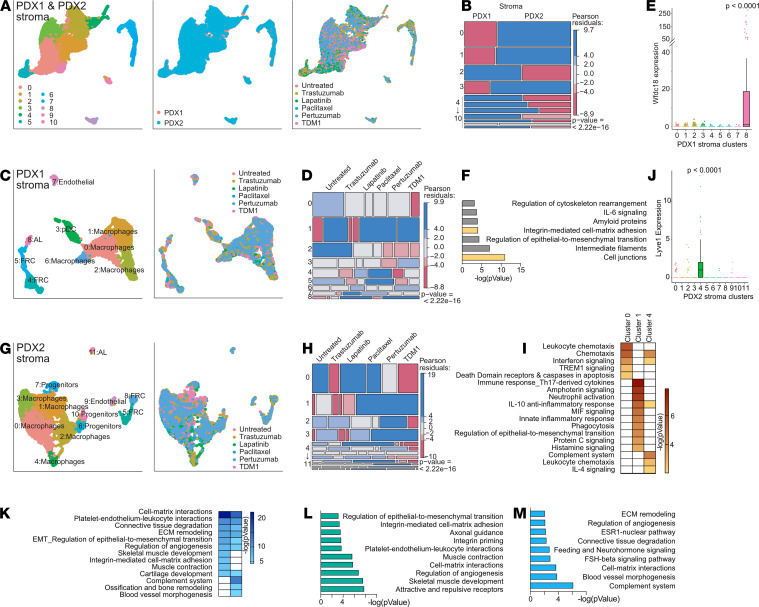
Stromal cell analysis by scRNA-Seq reveals distinct cell types contributing to differential drug response. (**A**) Combined analysis of stromal cells from *n* = 2 samples per each PDX and each condition (total 24 samples, average cell number per sample = 2169). UMAP plots colored by cluster (left panel), PDX (middle panel), and treatment (right panel). (**B**) Cell distribution among clusters based on PDX from which they were derived. (**C**) Analysis of stromal cells from PDX1. UMAP plots colored by cluster (left) and treatment (right). AL, alveolar luminal cells; pDC, plasmacytoid dendritic cells; FRCs, fibroblastic reticular cells. (**D**) Stromal cells from PDX1 distribution among clusters based on treatment. (**E**) Expression of Wfdc18 in different clusters of PDX1 stromal cells (log-normalized expression values). Unpaired 2-tailed Student’s *t* tests *P* value of comparison of cluster 8 to each of the other clusters is shown. (**F**) MetaCore GO Processes upregulated in AL cluster. (**G**) Analysis of stromal cells from PDX2. UMAP plots colored by cluster (left) and treatment (right). (**H**) Stromal cells from PDX2 distribution among clusters based on treatment. (**I**) GO Processes upregulated in 3 PDX2 macrophage clusters 0, 1, and 4. (**J**) Expression of Lyve1 in different clusters of PDX1 stromal cells (log-normalized expression values). Unpaired 2-tailed Student’s *t* tests *P* value of comparison of cluster 4 with each of the other clusters. (**K**) GO processes upregulated in 2 PDX2 FRC clusters 5 and 8. (**L**) GO processes upregulated in cluster 5 compared with cluster 8. (**M**) GO processes upregulated in cluster 8 compared with cluster 5. (**B**, **D**, and **H**) Red color, lower than expected frequency; blue, higher than expected. *P* value of χ^2^ test is shown.

Next, we explored differences in stromal composition between the 2 PDX and all treatment groups. We found that on average, 57.34% of the single cells extracted from the tumors were of murine origin (Supplemental Table 9). This set of cells enabled us to characterize the changes in stromal cell populations associated with differential treatment response. Since the clustering of cells based on scRNA-Seq is driven by cell type–specific programs, we expected to identify similar cell types associated with the tumor microenvironment of the 2 PDX models studied. However, combined analysis of the stroma from PDX1 and PDX2 revealed that the contribution of each PDX to the 10 identified clusters is mutually exclusive (Figure 6, A and B; and Supplemental Figure 13). This observation and the differential responses to treatment prompted us to analyze the stroma of the 2 PDXs independently (Figure 6, C, D, G, and H).

The analysis of cell cycle signature distribution across the stromal cells revealed a heterogeneous pattern, with cells in different cell cycle stages scattered over the clusters (Supplemental Figure 14). Thus, cell identity and/or expression programs are dominant over cell cycle programs in tumor stromal cells. Unlike in tumor cells, where different treatments did not affect the cell cycle, in the case of stroma cells from both PDXs, the frequencies of cells in G1 and S phase changed upon treatment. T-DM1, which was initially effective for both PDXs, depleted S phase (*P* = 4.88 × 10^9^) and increased G1 phase (*P* = 4.12 × 10^7^) stromal cell frequency, suggesting a higher degree of response within T-DM1–affected stroma.

To investigate mechanisms of acquired resistance in the stroma, we focused on clusters in which cell distribution was altered by paclitaxel and T-DM1, the treatments that affected the growth of PDX1 (Figure 6D and Supplemental Table 11). The stroma of the residual tumors after paclitaxel and T-DM1 treatment had higher frequencies of cells in cluster 8 (*P*_paclitaxel_ = 1.79 × 10^7^, *P*_TDM1_ = 1.61 × 10^8^). ImmGen analysis and an additional literature search for top overexpressed markers identified these cells as alveolar epithelial cells, expressing high levels of WAP four-disulfide core domain 18 (Wfdc18) (29) (Figure 6E and Supplemental Figure 13). Functionally, these mammary alveolar epithelial cells seem to exert immune regulation based on MetaCore process network analysis of the genes overexpressed in cluster 8 (Figure 6F and Supplemental Table 11). Thus, the development of resistance to paclitaxel and T-DM1 in this xenograft model might be regulated by a small population of alveolar epithelial cells potentially via the induction of antitumor immune responses.

Stromal cells within PDX2 largely classified into similar cell types identified in PDX1 (Figure 6G and Supplemental Table 11), with several additional clusters emerging. The distribution of cells within the clusters was more variable with treatments compared with PDX1 (Figure 6H), and 3 distinct macrophage clusters were differentially affected by treatments in PDX2. Macrophages in cluster 0 were most similar to the adipose tissue macrophages profile in ImmGen database (Supplemental Figure 12 and Supplemental Table 11), and macrophages in clusters 0, 1, and 4 demonstrated distinct pathway activation (Figure 6I). The most distinct macrophage cluster, cluster 4, expressed high levels of lymphatic vessel endothelial hyaluronan receptor 1 (Lyve1) (Figure 6J), a marker of specialized perivascular macrophages involved in the maintenance of arterial homeostasis (30). Lyve1^+^ macrophages were among the clusters depleted in residual tumors treated with T-DM1 (*P* = 8.81 × 10^8^) and pertuzumab (*P* = 4.35 × 10^7^), the 2 most effective treatments for PDX2, compared with all other treatments and untreated controls (Figure 6H). The stroma of these tumors also had fewer proliferating progenitors (*P*_TDM1_ = 0.028, *P*_pertuzumab_ = 0.00476; cluster 7) and fewer cells in 2 clusters identified as FRCs (*P*_TDM1,5_= 5.41 × 10^8^, *P*_TDM1,8_ = 2.94 × 10^5^, *P*_pertuzumab,8_ = 0.0151; clusters 5 and 8). FRCs, normally found in lymph nodes, may exert pleiotropic immunomodulatory functions (31). In PDX2, the 2 different FRC populations took part in cell-matrix interactions and regulation of angiogenesis or antigen presentation, respectively (Figure 6, J–M). Altogether, our results show that specific subsets of cells in the tumor microenvironment facilitate acquired resistance to treatment.

## Discussion

Being able to predict which treatment would benefit a particular case of HER2^+^ breast cancer could impact a significant population of cancer patients, sparing them multiple rounds of treatment and decreasing the risk of relapse. Following our previous findings (9), we here set out to test whether HER2-targeted therapy could exert strong selective pressures on genetically distinct subpopulations of cancer cells. Our single-cell in situ analyses show that the frequency of cells with resistance-conferring *PIK3CA* mutation is higher in untreated samples of patients who did not respond to trastuzumab/chemotherapy combination, and in those patients, the subpopulations with *PIK3CA* mutation and *ERBB2* amplification expanded after treatment. These frequency changes were significant, yet our small sample size did not allow for assigning a threshold that could be used to predict the response to neoadjuvant HER2-targeting trastuzumab/chemotherapy or T-DM1/pertuzumab combinations. However, in our CycIF analysis of samples from the T-DM1 cohort, the frequency of HER2-expressing tumor cells correlated with response to treatment. Simultaneous single-cell mutation detection and accurate protein expression in the same cell in situ would be needed to integrate genetic and phenotypic heterogeneity measurements in intact tumors. It is possible that the lack of correlation between changes in genetic heterogeneity and response in our NOR cohort was due to nonuniform chemotherapy treatment. The only significant variable associated with worse outcome was the decrease in Mut-Amp cell clustering, found in only 1 case analyzed. This was the only tumor in which *PIK3CA*^mut^ cells were more dispersed after treatment, despite having increased frequencies. This result is similar to our previous finding and suggests *PIK3CA*-mutant cancer cells are more migratory. Our results point to the importance of comparing samples from the same tumor before and after therapy and suggest that spatial organization of resistance-conferring cells may be an important variable in predicting long-term survival. Indeed, treatment response in the T-DM1 cohort was associated with spatial features, as tumors with no-pCR had tumor cells intermixed with activated macrophages, suggesting their resistance to immune ques. Temporal analysis of changes exerted by treatment on heterogeneous tumors will be necessary to fully capture the intricacies of tumor evolution and understand its directionality.

Lack of response to HER2-targeted treatment is a significant clinical challenge. ITH increases the chances that some subpopulations of cancer cells within a tumor already possess the resistance traits allowing them to survive therapy. Heterogeneous tumors are also more likely to adapt to the selective pressure posed by treatment and acquire new genetic and epigenetic features conferring resistance. In this study, we modeled the resistance of HER2^+^*PIK3CA*^mut^ breast cancer using 2 PDXs. As expected, treatment with trastuzumab and lapatinib had little effect on the PDX growth. However, the preexisting resistance in the 2 PDX models was different for paclitaxel and pertuzumab. PDX1 resistance to pertuzumab might be explained by the high activity of ER pathways and WNT and Hedgehog signaling in the untreated tumor. Similarly, paclitaxel resistance was associated with elevated expression of genes involved in cell cycle progression. The PDXs acquired resistance to T-DM1 and either paclitaxel or pertuzumab; however, the changes in the tumor cells themselves were not as pronounced as the effects of treatments on tumor stroma.

Single-cell analysis of cancer cells from both PDXs revealed increased frequency of a subpopulation able to activate an immune response, stress response, cell motility, and cell cycle after T-DM1 treatment. Moreover, the acquired resistance-related changes were prevalent in the tumor stroma. In PDX1, the resistance to paclitaxel and T-DM1 was linked to higher-than-expected numbers of alveolar epithelial cells. These murine cells most likely constitute the luminal fraction lining the mammary ducts, as they express luminal cytokeratins. Our results showed that major pathways activated in these cells were related to regulation of immune response. This work is the first report to our knowledge of crosstalk between breast cancer cells and normal mammary epithelium in the development of resistance and immunomodulation. Since our study was performed in an immunocompromised animal model, it is possible that other immune cells, including B and T cells, would also participate in this crosstalk. Further studies with on-treatment biopsies or longitudinal analysis of the tumor microenvironment during treatment will be needed to address the causal relationship between these cell types in acquired resistance.

Although the same types of stromal cells were identified in PDX2, the acquired resistance to T-DM1 and pertuzumab affected frequencies of 3 distinct stroma cell populations. Depletion of Lyve1^+^ macrophages was previously shown to increase arterial stiffness and collagen deposition around blood vessels, leading to arterial dysfunction (30). This may lead to inadequate blood flow, limiting further drug distribution to the tumor and creating a protumorigenic hypoxic microenvironment. FRCs in lymphoid organs control migration and positioning of immune cells and support homeostasis and immune activation (32). However, in our scRNA-Seq data, these cells, present within the tumor microenvironment and displaying a proangiogenic phenotype, were depleted in T-DM1 and pertuzumab-resistant tumors. Thus, acquisition of resistance in PDX2 seems to rely on limiting the delivery of the drugs to the tumor controlled by stroma cells and upregulation of hypoxia and stress response pathways in the tumor cells.

Our study provides detailed insight into the preexisting and acquired resistance of HER2^+^ PDX models to HER2-targeted therapy. The molecular and cellular profiles associated with resistance identified in this study merit thorough comparison with transcriptome and exome profiling from clinical trials of HER2-targeted agents. As more of these techniques are incorporated into clinical practice and larger data sets become available, further studies will be possible to test whether a combination of vascular normalization and immunotherapy could prevent the onset of resistance in HER2^+^ breast cancer.

## Methods

### Human tissue samples.

The *Norwegian cohort* comprised 30 cases of HER2^+^ breast cancer, with matched untreated biopsy and post-neoadjuvant treatment sample. The Response Evaluation Criteria In Solid Tumors (33) were used to score the effect of the neoadjuvant treatment, with pCR defined as no invasive tumor cells in the primary tumor region or lymph nodes after neoadjuvant treatment. No-pCR was defined as the presence of residual invasive tumor cells in primary tumor region or lymph nodes. In this cohort, 11 patients had a complete response, 13 had a partial response 1, 2 had partial response 2, and 2 had stable disease after neoadjuvant combination of chemotherapy and trastuzumab. The details of adjuvant treatment are shown in Supplemental Figure 1. The *T-DM1 cohort* comprised untreated FFPE tumor sections from 20 patients with *HER2*^+^ breast cancer from the ClinicalTrials.gov NCT02326974 clinical trial. For each case 2 different blocks with spatially distinct diagnostic biopsies were used. Pathological response to neoadjuvant treatment was reported using the Residual Cancer Burden calculator (34). Since this trial is ongoing, long-term responses are not yet available.

### PDXs.

PDX1 and PDX2 were derived from human HER2^+^ breast cancer at Institut Curie, Paris. PDXs were in accordance with published protocols.

PDX1 (HBCx-91) was derived from a 55-year-old woman with HER2^+^, ER^–^, and progesterone receptor–negative (PR^–^) breast cancer undergoing mastectomy. The patient received adjuvant FEC100 (3 cycles), taxol/trastuzumab (3 cycles), and trastuzumab (1 year). No relapse was present in 2020. PDX2 (HBCx-58) was derived from a mastectomy sample from a 90-year-old woman with HER2^+^ER^+^PR^–^ breast cancer after neoadjuvant Navelbin/trastuzumab (6 cycles; partial response). Postsurgery treatment included radiotherapy, letrozole/trastuzumab, and after metastatic spread occurred, trastuzumab monotherapy. The patient succumbed to the disease 32 months after the original diagnosis.

### STAR-FISH.

STAR-FISH for *PIK3CA* H1047R mutation was performed as described previously. The staining was imaged using a Leica SP5 scanning confocal microscope. *Z*-stacks from at least 3 areas of each sample were collected, and maximum projections were used to quantify signals in each individual nucleus (ImageJ macro, Supplemental Methods). Based on this quantification, each nucleus was assigned a genotype, and downstream analysis was performed with R v3.5.1 (https://www.R-project.org). For data analysis details see Supplemental Methods.

### Xenograft experiment.

Female NOG (NOD.Cg-*Prkdc^scid^ Il2rg^tm1Sug^*/JicTac) mice (Taconic) were used at 4–6 weeks of age. PDXs were expanded in NOG mice. A total of 2 million cells in 50% Matrigel (BD Biosciences) in DMEM (Gibco, Thermo Fisher Scientific), total volume 50 μL, were injected per mammary fat pad in a total volume 50 μL. In our experiments, we injected 30 animals with either PDX1 or PDX2, 2 contralateral fat pads per animal. Tumor diameter was monitored weekly. When all tumors reached over 0.5 cm diameter, the mice were randomized into 6 treatment groups. Paclitaxel (i.p.), T-DM1, trastuzumab, and pertuzumab (i.v) were delivered once weekly, while lapatinib was dosed by gavage 5 days a week (Supplemental Figure 4). Tumor diameter was measured on treatment weekly, and the experiment was terminated after 3 weeks of treatment. At the endpoint, tumors were extracted, weighed, and divided into 3 parts. One was immediately processed into a single-cell suspension (as described before, ref. 35), one was snap-frozen, and one was fixed in buffered formalin and processed into a paraffin block.

### Immunofluorescence staining.

FFPE tissues were baked overnight at 65°C, deparaffinized, and subjected to antigen retrieval in citrate buffer (pH 6; Invitrogen, Thermo Fisher Scientific) or Target Retrieval Solution (pH 9; Dako) for 20 minutes in a steamer. After blocking with 10% goat serum in 0.05% Tween-20 in PBS (PBST), the samples were incubated with primary antibody in 5% goat serum in PBST overnight at 4°C and washed 3 times for 5 minutes in PBST. Fluorescent dye–conjugated secondary antibodies’ incubation was held for 45 minutes at room temperature. The details regarding antibodies used are reported in Supplemental Table 12.

### CycIF.

FFPE tissues were mounted on adhesive slides and baked overnight at 55°C and an additional 30 minutes at 65°C. Tissues were deparaffinized with xylene and rehydrated with graded ethanol baths. Two-step antigen retrieval was performed in the Decloaking Chamber (Biocare Medical) using the following settings: set point 1 (SP1): 125°C, 30 seconds; SP2: 90°C, 30 seconds; SP limit: 10°C. Slides were further incubated in hot Target Retrieval Solution, pH 9 (Agilent, S236784-2), for 15 minutes. Slides were then washed in distilled H_2_O and once for 5 minutes in 1× PBS, pH 7.4. Sections were blocked in 10% normal goat serum (NGS, Vector Laboratories S-1000) and 1% bovine serum albumin (BSA, MilliporeSigma A7906) in PBS for 30 minutes at 20°C in a humid chamber, followed by PBS washes. Primary antibodies (Supplemental Table 12) were diluted in 5% NGS and 1% BSA in 1× PBS and applied overnight at 4°C in a humid chamber, covered with plastic coverslips (Bio-Rad, SLF0601). Following overnight incubation, tissues were washed 3 times for 10 minutes each in 1× PBS. Coverslips were mounted in Slowfade Gold plus DAPI mounting media (Life Technologies, Thermo Fisher Scientific, S36938). For details of image acquisition and analysis, see Supplemental Methods. For cell type determination and composition analysis, single-cell mean intensity was used, and threshold was assessed to generate a binary data set. Binary calls for each marker were combined into a gating strategy to define final cell types (Supplemental Table 3). For spatial analysis, nuclear centroids were used to calculate distances between cells. For each cell type A–cell type B pair, the number of cell type B within 75 μm of each single cell of type A was counted, and the mean number of cell type B proximal to cell type A was calculated for each case. For heterogeneity analysis, Shannon entropy of each case was calculated based on single-cell phenotype frequency of tumor receptor status expression (HER2^+^, ER^+^, HER2^+^ER^+^, HER2^–^ER^–^) or immune cell types (B cell, CD4^+^ T cell, CD8^+^ T cell, and CD68^+^ macrophage).

### exome-Seq and analysis.

Two independent tumors from each treatment group were used. DNA was extracted with QIAGEN Blood and Tissue Kit and shipped to Novogene for library construction and 100× coverage exome-Seq. Mutec1 (36) was used to call SNVs, and Strelka (37) and Mutec2 (38) were used to call insertions and deletions. This analysis was done in 2 ways: (a) a panel of normal samples from The Cancer Genome Atlas were used in place of matched controls to estimate absolute mutation levels in untreated and treated samples, and (b) the untreated samples were used as the matched controls for variant calling in the treatment samples to estimate relative changes compared with the untreated samples. Oncotator (39) and Variant Effect Predictor were used to annotate SNVs and indels. Variants appearing in noncancer variant annotations (e.g., dbSNP and 1,000 Genomes) were filtered out from downstream analysis. Finally, variants were filtered using Orientation Bias Filters, the MAF Panel of Normals Filter, and the BLAT Realignment Filter (40). The GATK3 CNV (38) pipeline was used to call copy number variants (CNVs), where the untreated samples were used in place of matched controls to call CNVs in the treated samples. R v3.5.1 (https://www.R-project.org) and Bioconductor v3.8 (41) were used for all downstream analyses. The list of CNVs was filtered to only include those with at least 100 reads. IRanges v2.16.0 (42) was used to identify CNVs with overlapping regions between the 2 replicates from each treatment group. CNVs with no overlaps in the other replicate were filtered out.

### RNA-Seq and analysis.

For bulk RNA-Seq, RNA was extracted from a snap-frozen tissue with QIAGEN Blood and Tissue Kit. Libraries were prepared using Illumina kit and sequenced according to standard protocol.

Demultiplexing and alignment of data were performed as previously described (35). To remove mouse contamination, reads that mapped uniquely to the human genome were kept for further analysis. A subset of these also mapped uniquely to the mouse genome, for which a threshold was determined for the minimum difference in alignment scores between human and mouse. The thresholds were determined, for each sample separately, by repeatedly taking random samples of the reads that mapped uniquely to both genomes, specified by varying alignment score differences, and statistically comparing sample alignment scores between human and mouse genomes using a 2-tailed *t* test. The minimum difference by which samples confidently displayed *P* ≤ 0.01 defined the threshold, beyond which reads were kept. Read counts for individual genes were generated using the htseq-count script of the HTSeq framework (version 0.6.1p1) (43) using modified parameters (--stranded no) and the hg19/mm10 refGene annotation file available at the UCSC Genome. The DESeq2 (44) R package version 1.16.1 was used to generate differential expression gene lists, with a fold change value ≥ 2 and *P*_adj_ ≤ 0.05. Pathway enrichment analysis was performed using MetaCore from Clarivate Analytics. Alternative splicing analysis was performed using LeafCutter v0.2.9 (45). GENCODE v19 (46) (GRCh37) was used to annotate exons and splice junctions.

### scRNA-Seq and data analysis.

Single-cell suspensions from 2 independent tumors were used from each xenograft and treatment group. Cell and library preparation for scRNA-Seq was performed according to 10x Genomics Chromium v2 protocol, targeting 2000 cells per sample. Cell Ranger v3.0.2 (47) was used to preprocess the scRNA-Seq output. cellranger mkfastq was used to demultiplex the base call files into FASTQ files; cellranger count was used to align and filter the FASTQ files individually, aligning to both the human (hg19) and mouse (mm10) genomes, and to generate feature-barcode matrices; cellranger aggr was used to normalize the separate runs to the same sequencing depth and aggregate the samples into 1 combined feature-barcode matrix. All together this resulted in separate feature-barcode matrices for stroma and tumor cells for each PDX model and replicate. R v3.5.1 (https://www.R-project.org) and Seurat v3 (48) were used for all downstream analysis. The data were filtered to include cells with at least 500 expressed genes and with a maximum of 25% of expressed genes coming from mitochondrial genes. The numbers of cells before and after filtering are shown in Supplemental Table 9. Cell cycle scores were assigned to each cell based on expression of G2/M and S phase markers using CellCycleScoring, using the marker gene list “cc.genes.updated.2019” as provided by Seurat. Data were normalized using SCTransform. The percentage of mitochondrial RNA per cell was regressed out during normalization, and default values were used for all other parameters. Samples were integrated using SelectIntegrationFeatures, PrepSCTransform, FindIntegrationAnchors, and IntegrateData. The number of features, nfeatures, was set to 3000, and the default values were used for all other parameters. The resulting normalized data were used for all downstream analyses. RunPCA was used for principal component analysis dimensionality reduction, and RunUMAP was used for UMAP dimensionality reduction. The first 10 principal components were used for both t-distributed stochastic neighbor embedding and UMAP. FindNeighbors was used to construct a shared nearest neighbor (SNN) graph, with k = 20. FindClusters was used to identify clusters based on the constructed SNN graph. A range of resolution values from 0.1 to 1 was tested to determine the appropriate number of clusters. Clustree v0.4.3 (49) was used to visualize the resulting clustering tree and investigate how clusters at different resolutions are related to each other. FindAllMarkers was used to find the differentially expressed genes for each of the clusters. The resulting gene lists were filtered to include only genes with adjusted *P* value less than 0.05. Pathway enrichment analysis was performed using MetaCore from Clarivate Analytics. The associations between treatment and cluster, cell cycle phase and cluster, and cell cycle phase and treatment were visualized using the mosaic function in vcd v1.4.4 (50). The size of each square in the mosaic plot represents the number of cells that fall into that respective category. The colors represent the Pearson residuals for each category in the contingency table, where a large value suggests greater frequency and a small value suggests smaller frequency than would be expected under the null hypothesis of independence. These Pearson residuals were used to compute separate *P* values for each category. The *P* value shown on the plot is computed using a χ^2^ test of independence and represents the significance of the overall association between the rows and columns.

### Data and biological material availability.

RNA-Seq, scRNA-Seq, and exome-Seq data have been deposited to the NCBI’s Gene Expression Omnibus database with accession number GSE161423. Raw STAR-FISH and CycIF analysis files are available on GitHub (https://github.com/mjaniszewska-lab/HER2_heterogeneity_2021; commit ID 04d6e0e). Availability of the human samples used in this study may be limited (requests should be addressed to HGR and IEK). PDXs are available upon request from EM.

### Statistics.

Sample size for mouse experiments was determined based on prior studies and pilot experiments to ensure that sufficient power could be obtained (51). Statistical analyses were performed with GraphPad Prism software or R. Box-and-whisker plots show mean (midline), 25th–75th percentile (box), and 5th–95th percentile (whiskers). For 2-tailed Student’s *t* test, a *P* value of less than 0.05 was considered significant. Other statistical tests used in this study were Wilcoxon rank-sum test, Kruskal-Wallis test, χ^2^ calculation, log-rank test, and Cox regression.

### Study approval.

All experiments with use of human tumor tissue were approved by the Dana-Farber Cancer Institute (DFCI) IRB and performed according to DFCI protocols 14-400 and 16-233. For the Norwegian cohort study, informed and written consent was obtained from all patients, and the study was approved by the Regional Ethical Committee (South-east of Norway; Oslo, Norway; no. S-06495b). The study methodologies conformed to the standards set by the Declaration of Helsinki. PDXs were established with informed consent from the patients and with the approval of the French Ethics Committee (project authorization no. 02163.02; Paris, France). All animal procedures were approved by DFCI Animal Care and Use Committee and performed at DFCI according to DFCI protocol 11-023.

## Author contributions

MJ and SS are co–first authors, with MJ listed first since she designed and comanaged the project with KP and FM. MJ performed xenograft, molecular profiling, and immunohistochemical experiments and data analyses. SS analyzed RNA-Seq, scRNA-Seq, STAR-FISH, and exome-Seq data. NWH and BO analyzed RNA-Seq data. JE, EB, ZH, and KC performed CycIF experiments and data analyses. NLK and KCM assisted with immunohistochemical staining. MA helped with scRNA-Seq libraries’ generation. AT helped with exome-Seq data analysis. SC helped with scRNA-Seq analysis. OMF, EPW, and IEK provided clinical samples from the T-DM1 trial and consulted on xenograft assay outcomes. IHR and HGR provided clinical samples from Norwegian cohort. EM provided the PDX models. KP supervised with help from FM, PTS, and JWG. All authors helped to design the study and write the manuscript.

## Supplementary Material

Supplemental data

Supplemental Table 1

Supplemental Table 2

Supplemental Table 3

Supplemental Table 4

Supplemental Table 5

Supplemental Table 6

Supplemental Table 7

Supplemental Table 8

Supplemental Table 9

Supplemental Table 10

Supplemental Table 11

Supplemental Table 12

## Version 1 04/22/2021

In-Press Preview

## Version 2 06/08/2021

Electronic publication

## References

[1] Russnes HG (2017). Breast cancer molecular stratification: from intrinsic subtypes to integrative clusters. Am J Pathol.

[2] Slamon D (2011). Adjuvant trastuzumab in HER2-positive breast cancer. N Engl J Med.

[3] Marti JLG (2020). The evolving landscape of HER2-directed breast cancer therapy. Curr Treat Options Oncol.

[4] Rinnerthaler G (2019). HER2 directed antibody-drug-conjugates beyond T-DM1 in breast cancer. Int J Mol Sci.

[5] Yang SX (2016). New insights on PI3K/AKT pathway alterations and clinical outcomes in breast cancer. Cancer Treat Rev.

[6] de Melo Gagliato D (2016). Mechanisms of resistance and sensitivity to anti-HER2 therapies in HER2+ breast cancer. Oncotarget.

[7] Rye IH (2018). Intratumor heterogeneity defines treatment-resistant HER2+ breast tumors. Mol Oncol.

[8] Zhang H (2020). Applying the new guidelines of HER2 testing in breast cancer. Curr Oncol Rep.

[9] Janiszewska M (2015). In situ single-cell analysis identifies heterogeneity for PIK3CA mutation and HER2 amplification in HER2-positive breast cancer. Nat Genet.

[10] Lewis Phillips GD (2008). Targeting HER2-positive breast cancer with trastuzumab-DM1, an antibody-cytotoxic drug conjugate. Cancer Res.

[11] Groenendijk FH, Bernards R (2014). Drug resistance to targeted therapies: déjà vu all over again. Mol Oncol.

[12] Pernas S, Tolaney SM (2019). HER2-positive breast cancer: new therapeutic frontiers and overcoming resistance. Ther Adv Med Oncol.

[13] Kim MS (2010). Somatic mutations and losses of expression of microRNA regulation-related genes AGO2 and TNRC6A in gastric and colorectal cancers. J Pathol.

[14] Sidor C (2019). Mask family proteins ANKHD1 and ANKRD17 regulate YAP nuclear import and stability. Elife.

[15] Deng M (2009). Identification and functional analysis of a novel cyclin e/cdk2 substrate ankrd17. J Biol Chem.

[16] Oh E (2017). Inhibition of ubiquitin-specific protease 34 (USP34) induces epithelial-mesenchymal transition and promotes stemness in mammary epithelial cells. Cell Signal.

[17] Winter SF (2012). Allelic variation and differential expression of the mSIN3A histone deacetylase complex gene Arid4b promote mammary tumor growth and metastasis. PLoS Genet.

[18] Luo LY (2002). Higher expression of human kallikrein 10 in breast cancer tissue predicts tamoxifen resistance. Br J Cancer.

[19] Xi Z (2004). Kallikrein 4 is associated with paclitaxel resistance in ovarian cancer. Gynecol Oncol.

[20] Azenshtein E (2002). The CC chemokine RANTES in breast carcinoma progression: regulation of expression and potential mechanisms of promalignant activity. Cancer Res.

[21] Walens A (2019). CCL5 promotes breast cancer recurrence through macrophage recruitment in residual tumors. Elife.

[22] Araujo JM (2018). Effect of CCL5 expression in the recruitment of immune cells in triple negative breast cancer. Sci Rep.

[23] Allaoui R (2016). Cancer-associated fibroblast-secreted CXCL16 attracts monocytes to promote stroma activation in triple-negative breast cancers. Nat Commun.

[24] Whitesell L (2014). HSP90 empowers evolution of resistance to hormonal therapy in human breast cancer models. Proc Natl Acad Sci U S A.

[25] Post TW (1991). Membrane cofactor protein of the complement system: alternative splicing of serine/threonine/proline-rich exons and cytoplasmic tails produces multiple isoforms that correlate with protein phenotype. J Exp Med.

[26] Hansen AS (2016). Non-random pairing of CD46 isoforms with skewing towards BC2 and C2 in activated and memory/effector T cells. Sci Rep.

[27] Wang Y (2010). Differential functions of growth factor receptor-bound protein 7 (GRB7) and its variant GRB7v in ovarian carcinogenesis. Clin Cancer Res.

[28] Park S (2019). Differential functions of splicing factors in mammary transformation and breast cancer metastasis. Cell Rep.

[29] Robinson GW (1995). Mammary epithelial cells undergo secretory differentiation in cycling virgins but require pregnancy for the establishment of terminal differentiation. Development.

[30] Lim HY (2018). Hyaluronan receptor LYVE-1-expressing macrophages maintain arterial tone through hyaluronan-mediated regulation of smooth muscle cell collagen. Immunity.

[31] Perez-Shibayama C (2019). Fibroblastic reticular cells at the nexus of innate and adaptive immune responses. Immunol Rev.

[32] Fletcher AL (2020). The fibroblastic T cell niche in lymphoid tissues. Curr Opin Immunol.

[33] Nishino M (2010). New response evaluation criteria in solid tumors (RECIST) guidelines for advanced non-small cell lung cancer: comparison with original RECIST and impact on assessment of tumor response to targeted therapy. AJR Am J Roentgenol.

[34] Symmans WF (2007). Measurement of residual breast cancer burden to predict survival after neoadjuvant chemotherapy. J Clin Oncol.

[35] Janiszewska M (2019). Subclonal cooperation drives metastasis by modulating local and systemic immune microenvironments. Nat Cell Biol.

[36] Cibulskis K (2013). Sensitive detection of somatic point mutations in impure and heterogeneous cancer samples. Nat Biotechnol.

[37] Saunders CT (2012). Strelka: accurate somatic small-variant calling from sequenced tumor-normal sample pairs. Bioinformatics.

[38] McKenna A (2010). The Genome Analysis Toolkit: a MapReduce framework for analyzing next-generation DNA sequencing data. Genome Res.

[39] Ramos AH (2015). Oncotator: cancer variant annotation tool. Hum Mutat.

[40] Carter SL (2012). Absolute quantification of somatic DNA alterations in human cancer. Nat Biotechnol.

[41] Huber W (2015). Orchestrating high-throughput genomic analysis with Bioconductor. Nat Methods.

[42] Lawrence M (2013). Software for computing and annotating genomic ranges. PLoS Comput Biol.

[43] Anders S (2015). HTSeq--a Python framework to work with high-throughput sequencing data. Bioinformatics.

[44] Love MI (2014). Moderated estimation of fold change and dispersion for RNA-seq data with DESeq2. Genome Biol.

[45] Li YI (2018). Annotation-free quantification of RNA splicing using LeafCutter. Nat Genet.

[46] Frankish A (2019). GENCODE reference annotation for the human and mouse genomes. Nucleic Acids Res.

[47] Zheng GX (2017). Massively parallel digital transcriptional profiling of single cells. Nat Commun.

[48] Stuart T (2019). Comprehensive integration of single-cell data. Cell.

[49] Zappia L, Oshlack A (2018). Clustering trees: a visualization for evaluating clusterings at multiple resolutions. Gigascience.

[50] Meyer D (2006). The strucplot framework: visualizing multi-way contingency tables with vcd. J Stat Softw.

[51] Marusyk A (2014). Non-cell-autonomous driving of tumour growth supports sub-clonal heterogeneity. Nature.

[52] Jenkinson G (2020). LeafCutterMD: an algorithm for outlier splicing detection in rare diseases. Bioinformatics.

