# Supplementary material for: The impact of tumor epithelial and microenvironmental heterogeneity on treatment responses in HER2+ breast cancer
Source: JCI Insight. 2021 Jun 8;6(11):e147617. doi: 10.1172/jci.insight.147617 (PMC8262355; doi:10.1172/jci.insight.147617)
Supplement: Supplemental data [file jciinsight-6-147617-s065.pdf]

SUPPLEMENTAL FIGURES

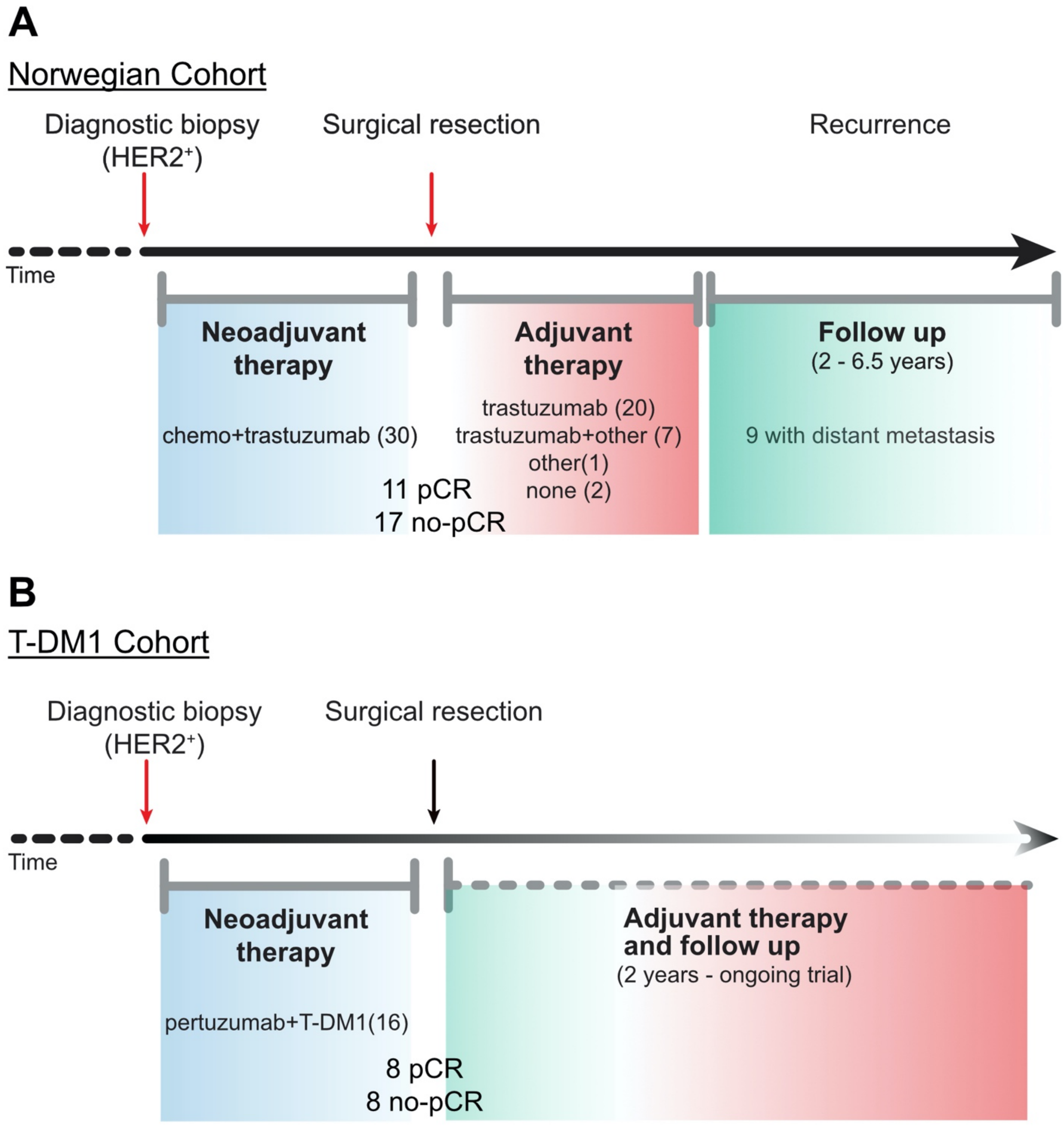

Supplemental Figure 1. HER2$^+$ breast cancer cohorts description. Patients are classified at the time of surgical resection as pCR - complete pathologic response, no-pCR – no complete pathologic response. (**A**) Norwegian cohort. Matched diagnostic biopsy (all cases) and surgical resection specimen (no-pCR cases only) were used in our study. All 30 patients received neoadjuvant chemotherapy combined with trastuzumab. Patients received adjuvant targeted therapy and long-term follow-up was reported. (**B**) T-DM1 cohort. Diagnostic biopsy was used in our study. All 16 patients received neoadjuvant pertuzumab and T-DM1. Long-term follow-up is not yet available (ongoing trial).

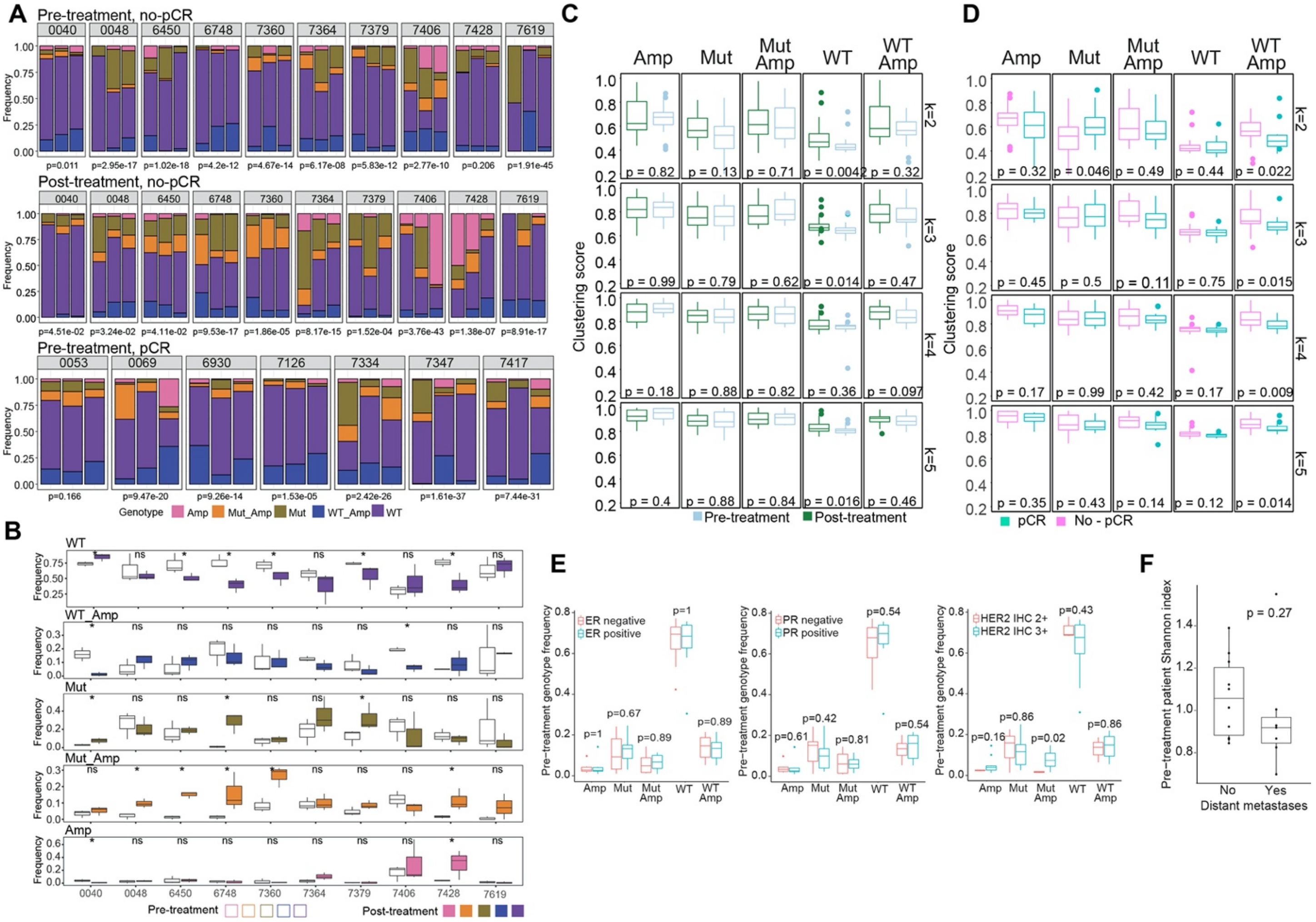

Supplemental Figure 2. Cellular genetic and spatial diversity in the Norwegian cohort. (**A**) Genotype frequencies of pre-treatment samples patients with pathological complete response (pCR) vs. no-pCR, and post-treatment samples from patients with no-pCR. Each bar represents an individual imaged area, grouped by patient ID. p values from likelihood of heterogeneity tests are shown. (**B**) Comparison of each genotype frequency in pre- (empty box) and post-treatment sample (filled box) for each individual patient. p values of Kruskal Wallis test * <0.05, ns – nonsignificant. (**C-D**), Spatial clustering of cells with different genotypes in pre- vs post-treatment samples (**C**) and in patients with pCR vs. no PCR (**D**). k means clustering was used and results for k=2, 3, 4 and 5 are shown. Box plot indicates the median and quantiles, whiskers indicate the min. and max. values. p values of Wilcoxon test are shown. (**E**) Pretreatment genotype frequencies in samples classified based on estrogen receptor (ER), progesterone receptor (PR), and HER2 immunohistochemistry status reported in clinical data reports in the NOR cohort. Box plot indicates the median and quantiles, whiskers indicate the min and max values. p values of Wilcoxon test are shown. IHC 2+ - medium expression of HER2, IHC 3+ - high expression of HER2. (**F**) Association between pre-treatment Shannon index and distant metastasis occurrence in NOR cohort. Each dot represents a case, box plot indicates the median and quantiles, whiskers indicate the min and max values. p value of Wilcoxon test is shown.

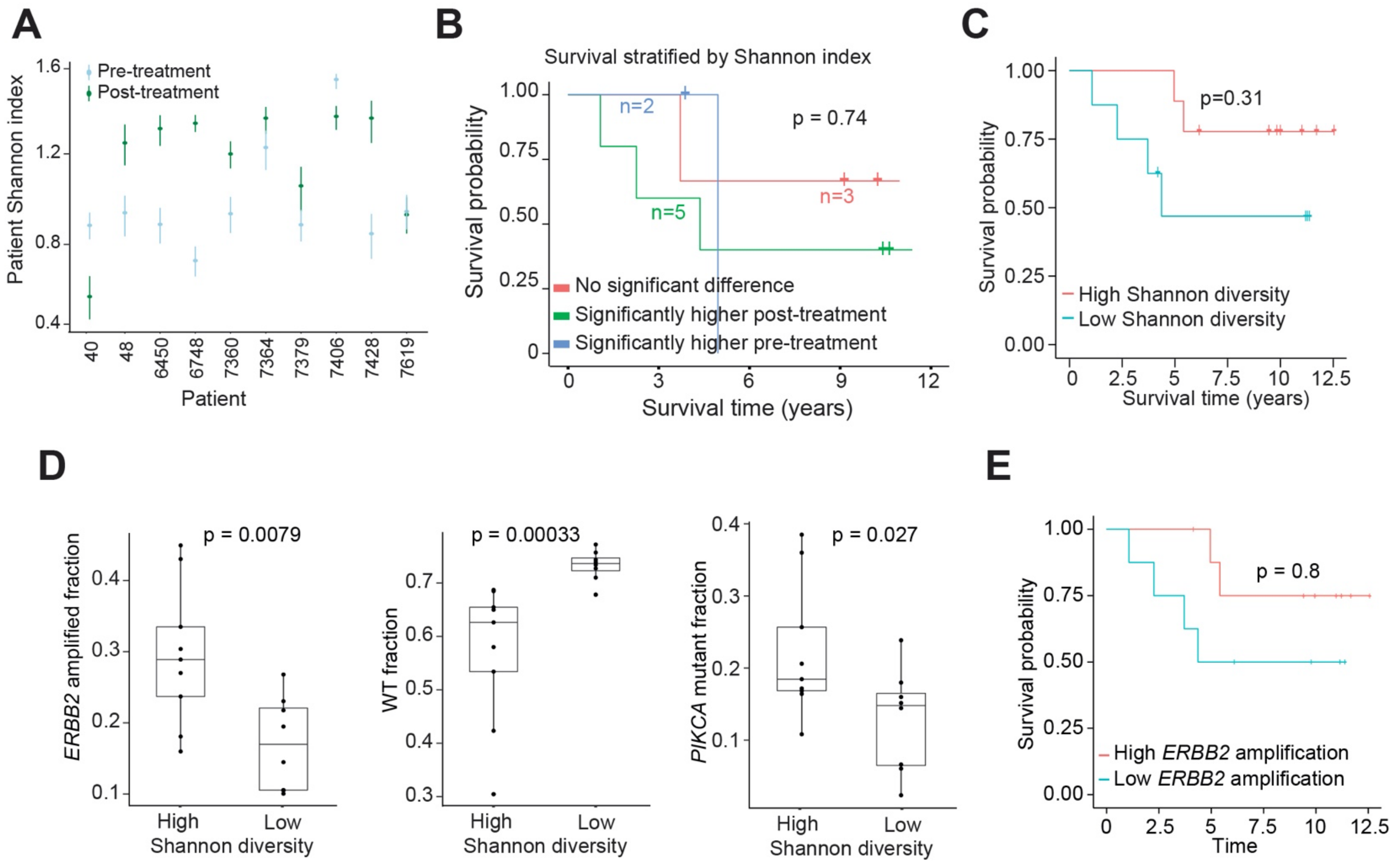

Supplemental Figure 3. Cellular genetic heterogeneity and clinic-pathologic data in the Norwegian cohort. (**A**) Bootstrap values of Shannon diversity index. Points represent the median resampled values; lines indicate the 95% confidence interval. (**B**) Kaplan-Meier plots of NOR cohort patient breast cancer-specific survival stratified by change in Shannon diversity index pre- and post-treatment. Log-rank test p values are shown. (**C**) Kaplan-Meier plot of NOR cohort patient survival stratified by median Shannon diversity index. Dichotomization is done for visualization only. The p-values shown are obtained from Cox regression done using the continuous heterogeneity scores. (**D**) Association between pre-treatment Shannon diversity index stratified by the median value and frequency of cells with *ERBB2* amplification (left), WT signal (middle), and with *PIKCA* mutation (right). The p-values shown are obtained from a Wilcoxon test. (**E**) Kaplan-Meier plot of NOR cohort patient survival stratified by median of *ERBB2* amplification. Dichotomization is done for visualization only. The p-values shown are obtained from Cox regression using the continuous *ERBB2* amplification.

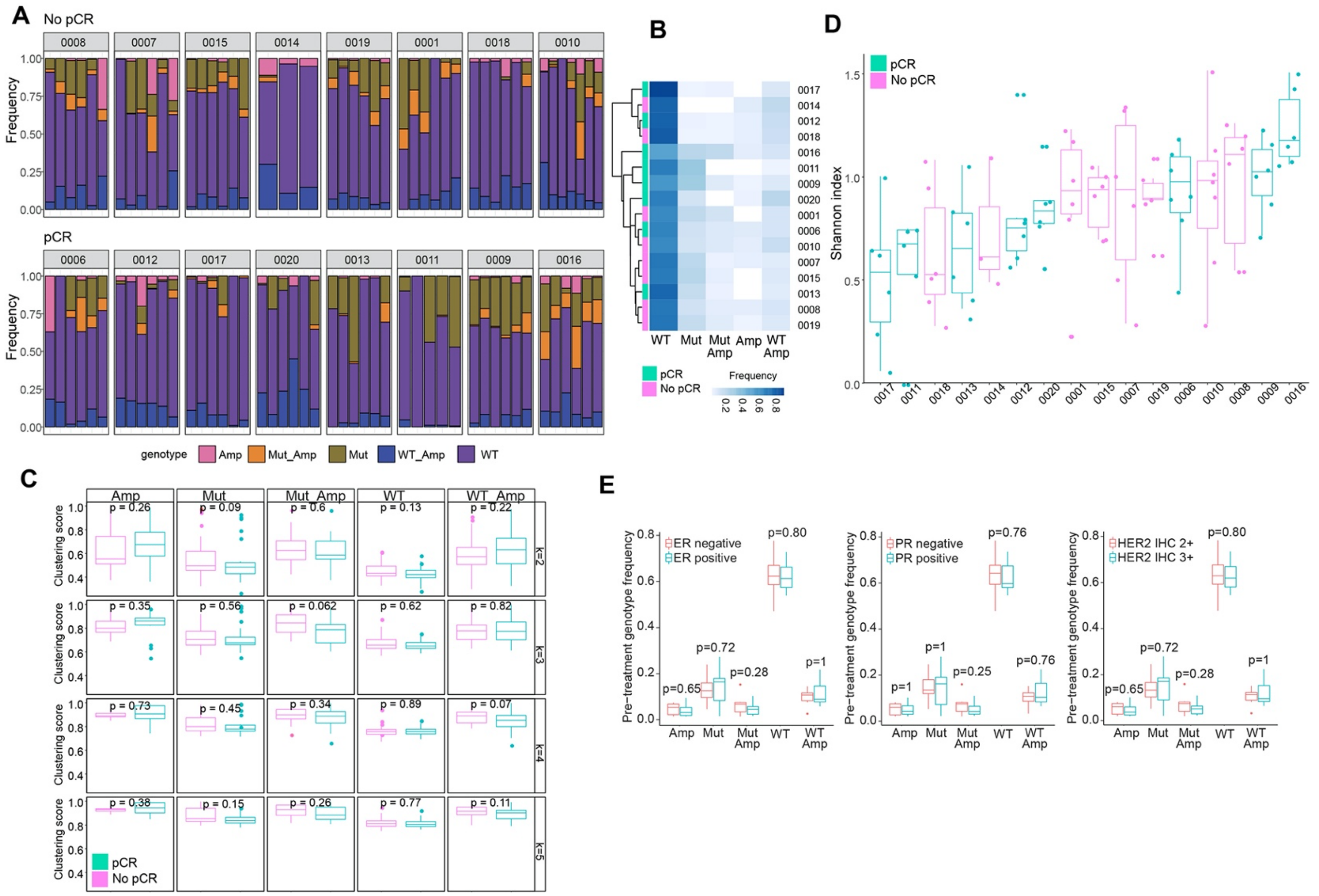

Supplemental Figure 4. T-DM1 cohort cellular genetic and spatial diversity. (**A**) Genotype frequencies. Each bar represents an individual imaged area, grouped by patient ID. (**B**) Unsupervised clustering of genotype frequencies per patient. Patients' response status indicated in green and magenta. (**C**) Spatial clustering of cells with different genotypes in patients with pCR vs. no-pCR. k means clustering was used and results for k=2, 3, 4 and 5 are shown. Box plot indicates the median and quantiles, whiskers indicate the min. and max. values. p values of Wilcoxon test are shown. (**D**) Shannon index of cellular genetic diversity for each patient. Each data point represents the index calculated for each imaged area separately, grouped per patient. Box plot indicates the median and quantiles. Colors indicate response to neoadjuvant therapy. (**E**) Pretreatment genotype frequencies in samples classified based on estrogen receptor (ER), progesterone receptor (PR), and HER2 immunohistochemistry status reported in clinical data reports in the T-DM1 cohort. Box plot indicates the median and quantiles, whiskers indicate the min and max values. p values of Wilcoxon test are shown. IHC 2+ - medium expression of HER2, IHC 3+ - high expression of HER2.

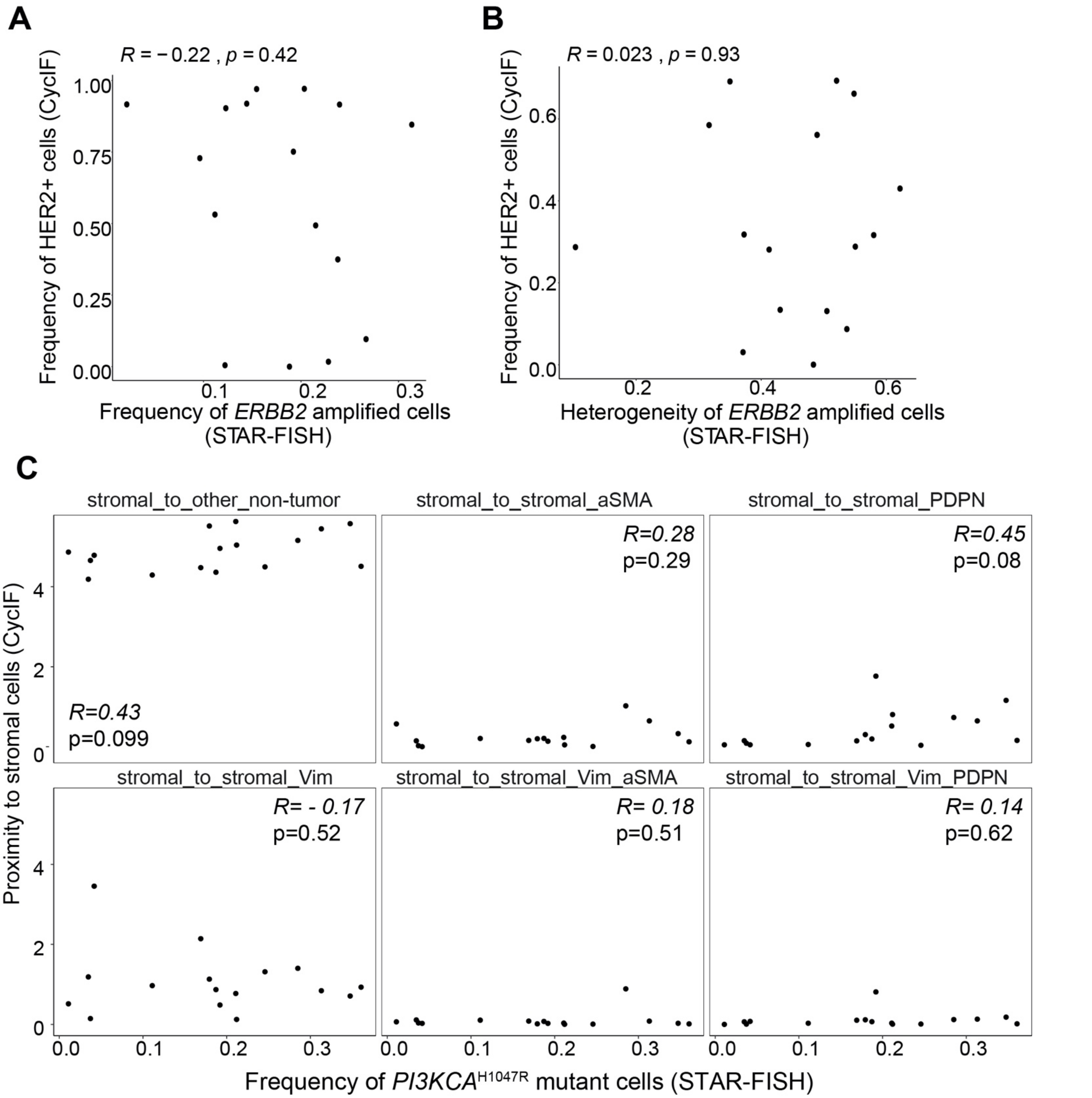

Supplemental Figure 5. Associations between cyclic immunofluorescence and STAR-FISH results in the T-DM1 cohort. (**A-B**) Correlation between the frequency of HER2+ cells assessed by CycIF with the frequency of *ERBB2* amplified cells measured by STAR-FISH (**A**) and sample heterogeneity quantified by the two methods (**B**). (**C**) Associations between *PIK3CA*^{H1047R} mutation frequency measured by STAR-FISH and stromal cell distribution assessed by CycIF.

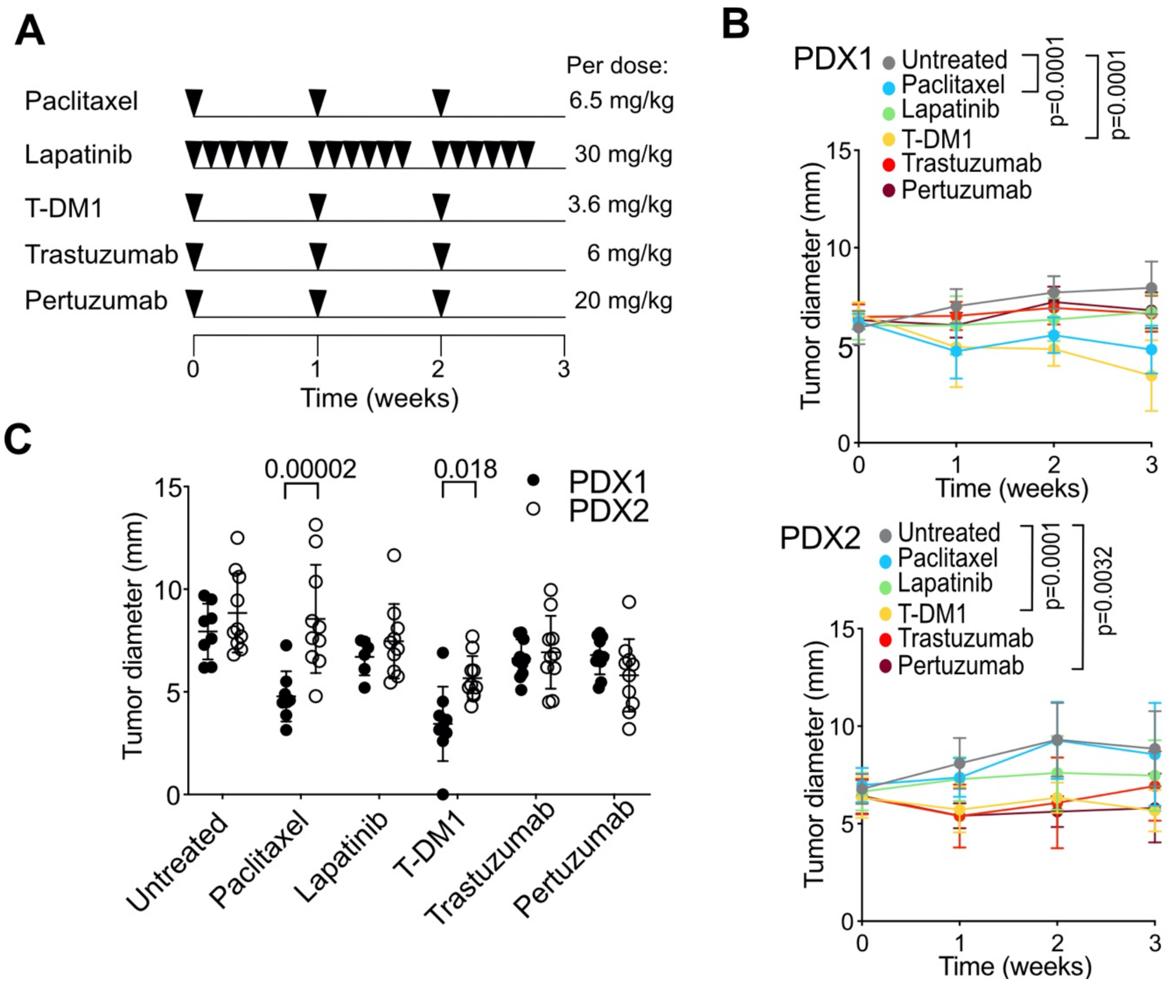

Supplemental Figure 6. PDX treatment dosing schedule and tumor growth kinetics. (**A**) Drug dosing schedule. (**B**) Tumor growth kinetics. Mean ± s.d. is shown; n=10 tumors and 5 animals per group. Significant adjusted p values of Dunnett's multiple comparison test are shown. (**C**) Tumor diameter at the endpoint of the in vivo experiment. Significant adjusted p values of unpaired two-tailed multiple t tests are shown.

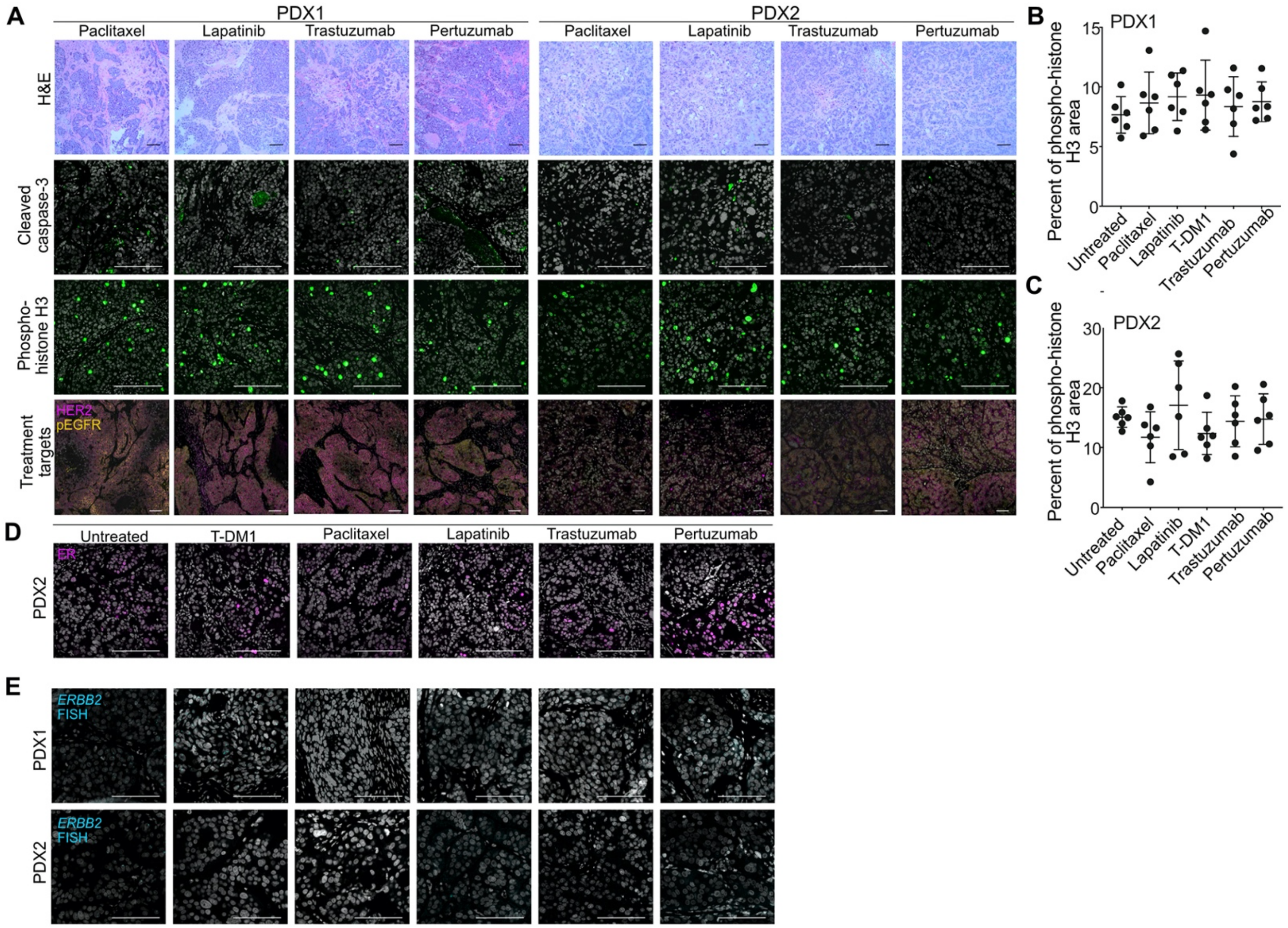

Supplemental Figure 7. Proliferation and apoptosis in residual tumors. (**A**) Representative images of the histology (hematoxylin-eosin staining; upper panels) and immunofluorescence staining for apoptosis marker (cleaved caspase-3), cell proliferation marker (phosphor-histone H3) and treatment targets (HER2 and phospho-EGFR proteins). Scale bars, 100um. Staining was repeated twice with similar results. (**B-C**) Quantification of phosphor-histone H3 immunostaining in PDX1 (**B**) and PDX2 (**C**). The area was normalized to nuclei count. Each data point represents different imaged area (n=3), two independent tumors per each sample were imaged (total n=6 per each experimental group). Mean ± s.d. is shown, unpaired two-tailed t-test results were non-significant for any treatment vs untreated controls comparison. (**D-E**), Representative images of immunofluorescence staining for estrogen receptor, expressed only in PDX2 (**D**) and fluorescence in situ hybridization (FISH) for *ERBB2* (**E**). Scale bars, 100um. Staining was repeated twice with similar results.

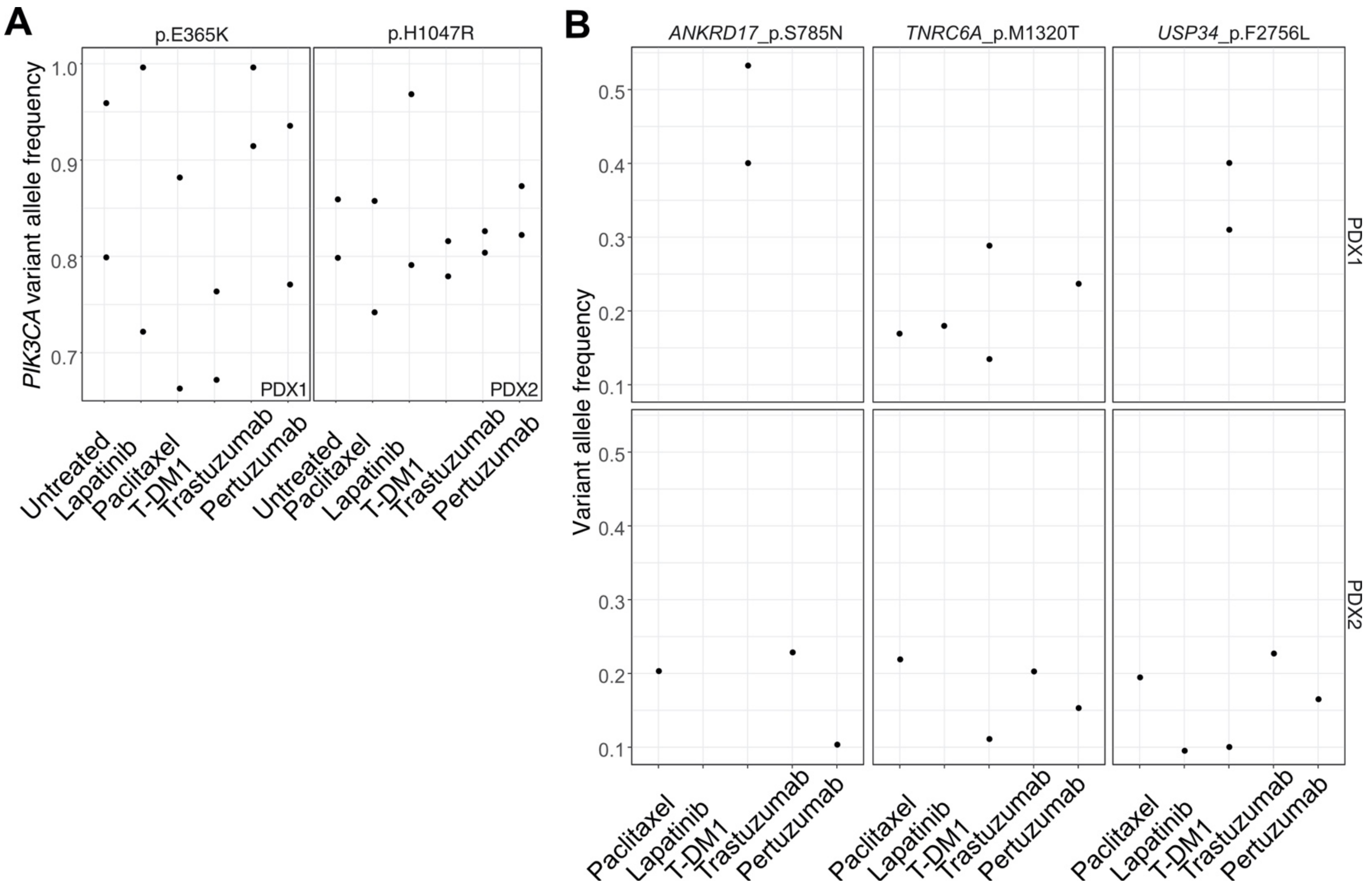

Supplemental Figure 8. Variant allele frequency in residual tumors. (**A**) *PIK3CA* mutation frequency in PDX1 for p.E365K site (left) and PDX2 for p.H1047R site (right) based on exome sequencing (n=2 per each treatment group). (**B**) Variant allele frequency of treatment-associated mutations. Untreated samples were used as a reference for mutation detection. Some mutations were detected in only one of two sequenced tumor samples.

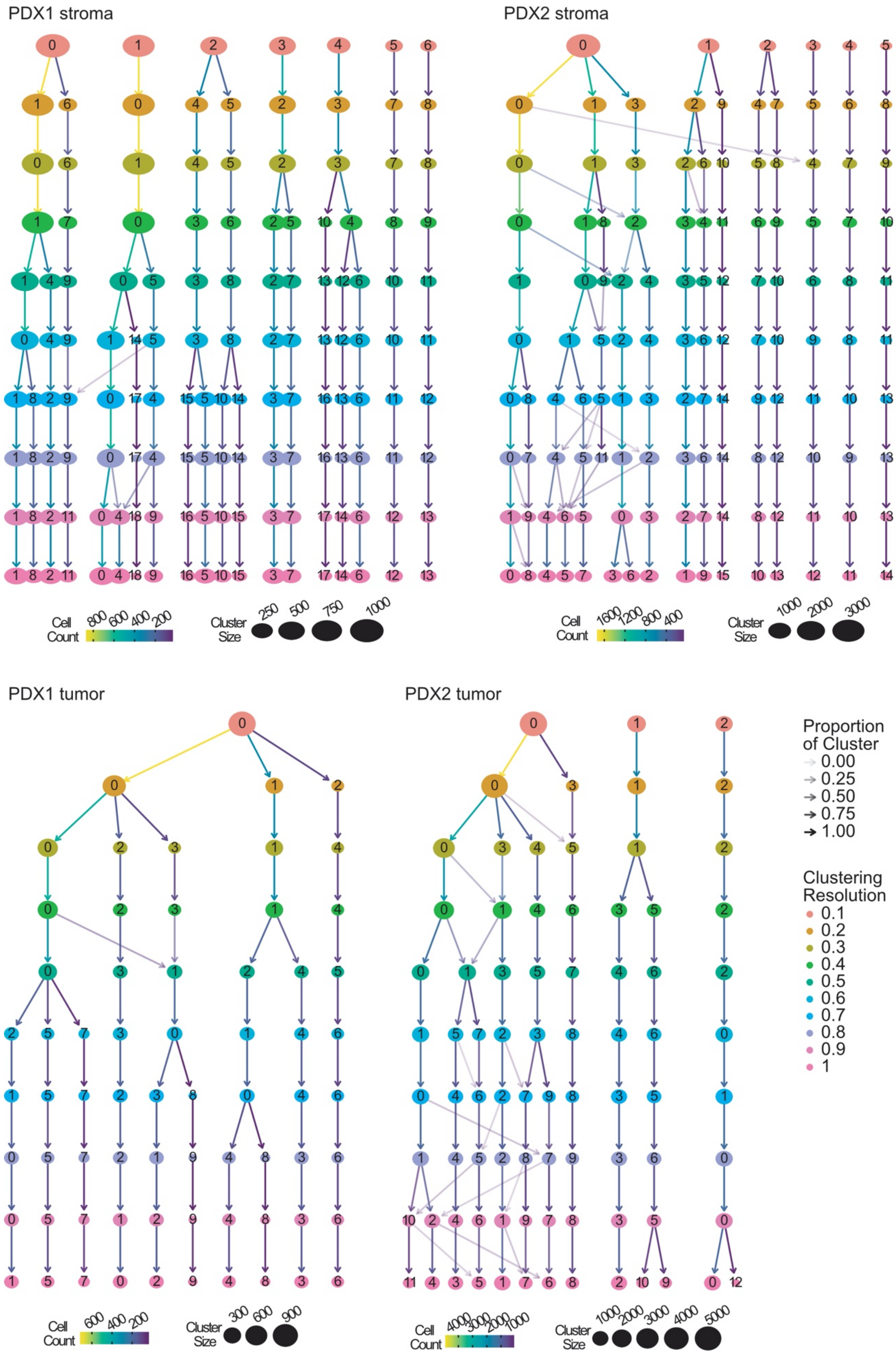

Supplemental Figure 9. Single cell data clustering dendrograms. Dendrograms of sctransform data clustering with increasing clustering resolution. Arrows indicate how each cluster is split after increasing the resolution by 0.1.

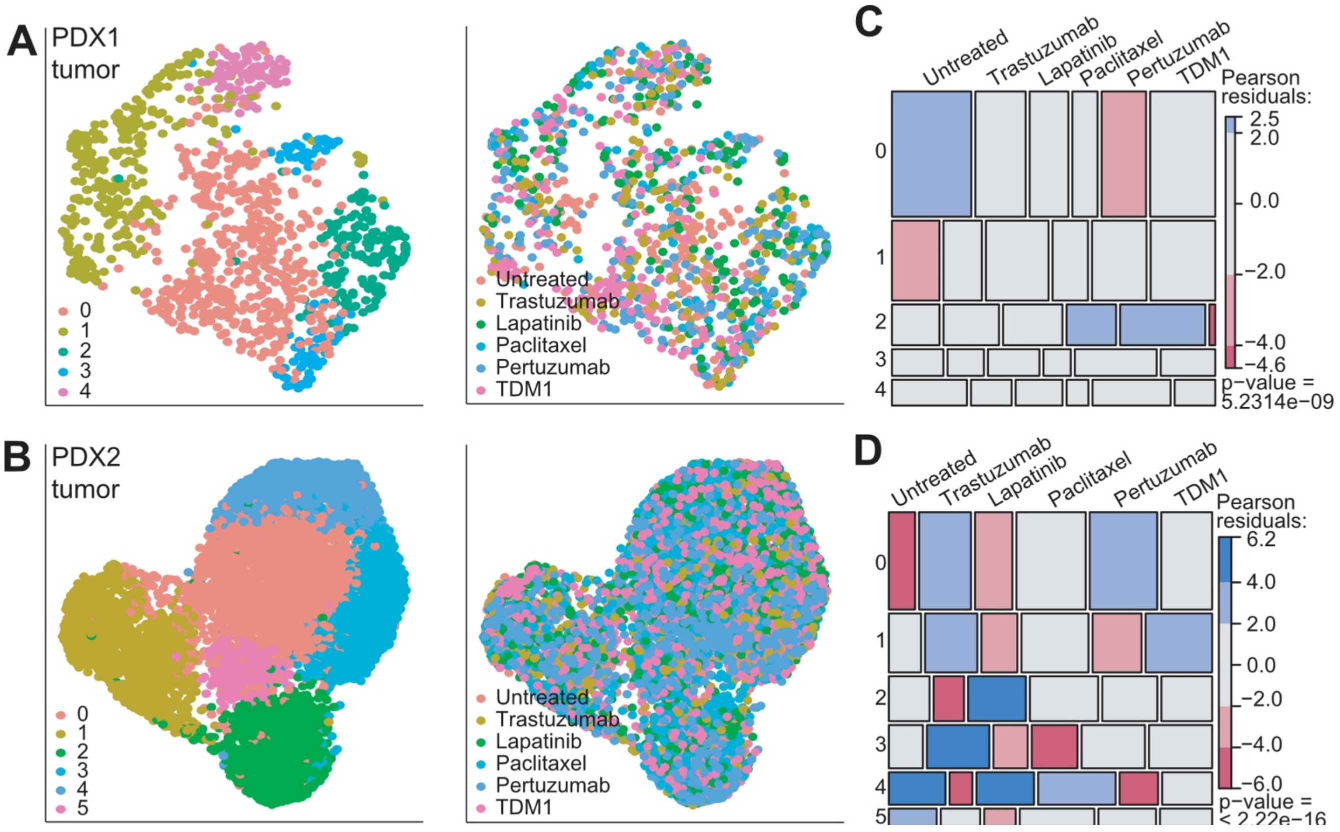

Supplemental Figure 10. Single cell expression profiling analysis of PDX1 and PDX2 tumor cells, independently. (**A-B**) UMAP clustering of PDX1 (**A**) and PDX2 (**B**) tumor cells colored by cluster (left) or by treatment (right). (**C-D**) Cell distribution among clusters depending on treatment of PDX1 (**C**) and PDX2 (**D**). Size of each box is proportional to number of cells within a cluster from given sample (treatment). Red color indicates lower than expected frequency, blue – higher than expected. P value of $\mathcal{X}^2$ test is shown.

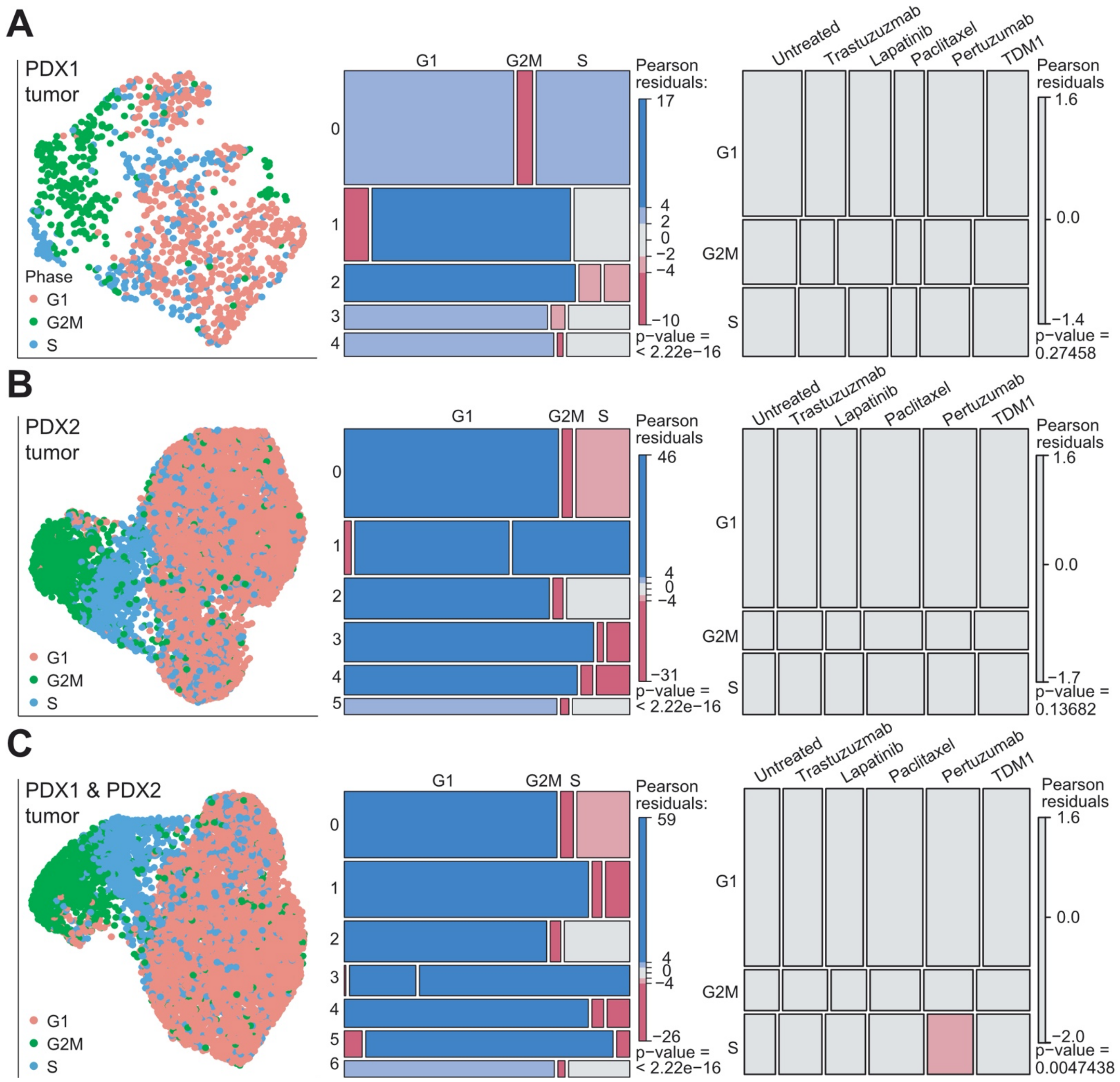

Supplemental Figure 11. Cell cycle gene expression analysis of PDX1 and PDX2 tumor cells. (**A-C**) UMAP clustering of PDX1 (**A**), PDX2 (**B**) or both PDXs combined (**C**). Tumor cells are colored according to the cell cycle phase (G1, G2M or S phase). Middle and right plots - cell distribution among clusters depending on cell cycle phase. Size of each box is proportional to number of cells within a cluster from given cell cycle phase (middle panel) or treatment (right panel). Red color indicates lower than expected frequency, blue – higher than expected. P value of $\mathcal{X}^2$ test is shown.

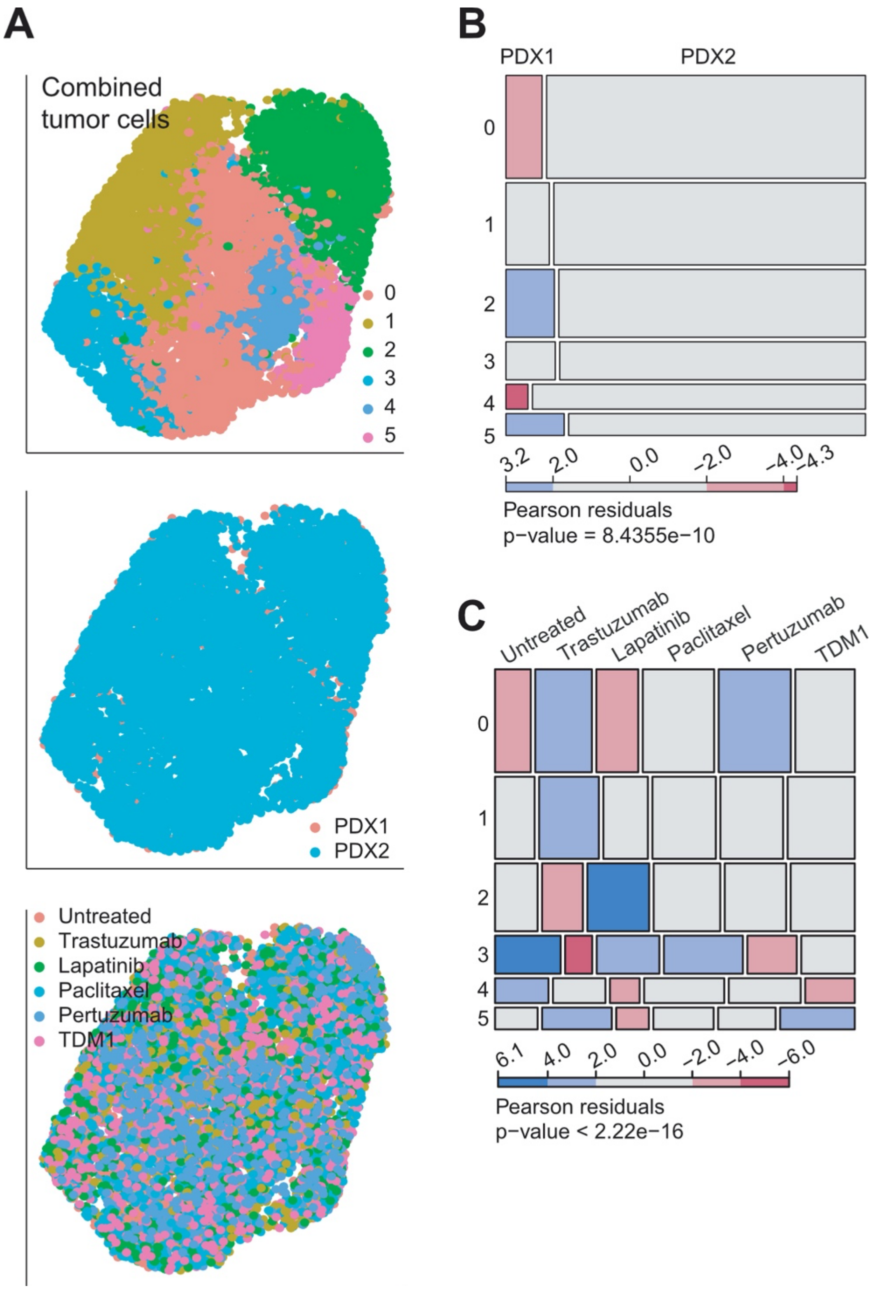

Supplemental Figure 12. Single cell expression profiling analysis of combined PDX1 and PDX2 tumor cells after regression of cell cycle genes. (**A**) UMAP clustering of combined PDX1 and PDX2 tumor cells colored by cluster (top panel), tumor of origin (middle panel) and treatment (bottom panel). (**B-C**), Cell distribution among clusters depending on tumor of origin (**B**) or treatment (**C**). Size of each box is proportional to number of cells within a cluster from given cell cycle phase (middle panel) or treatment (right panel). Red color indicates lower than expected frequency, blue – higher than expected. P value of $\mathcal{X}^2$ test is shown.

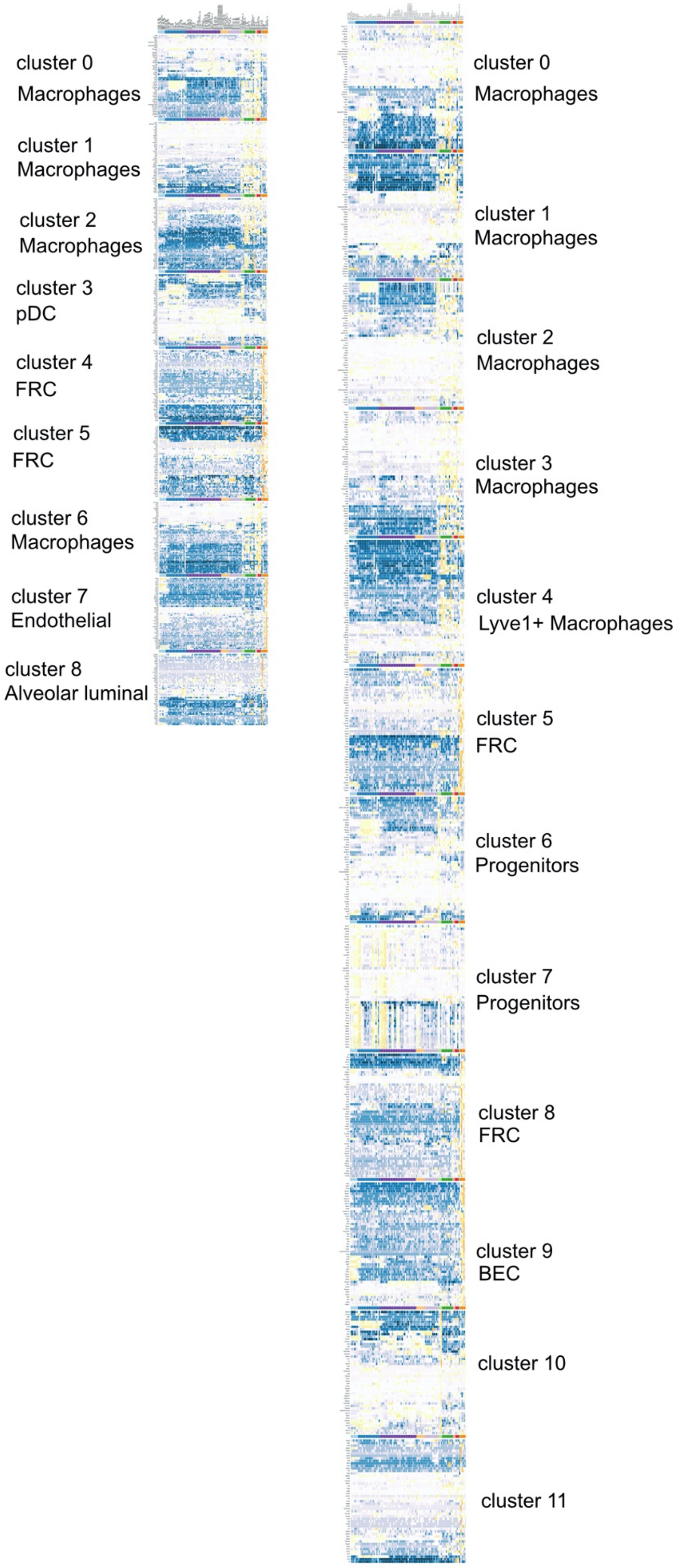

Supplemental Figure 13. Stroma cell type identification. Heat map represents the expression of the top 50 differentially expressed genes for each stromal cell cluster identified in our single cell RNA-seq experiment (columns) within the cell type-specific expression data from ImmGen consortium (rows). Upper panels – PDX1 stroma, lower panels – PDX2 stroma.

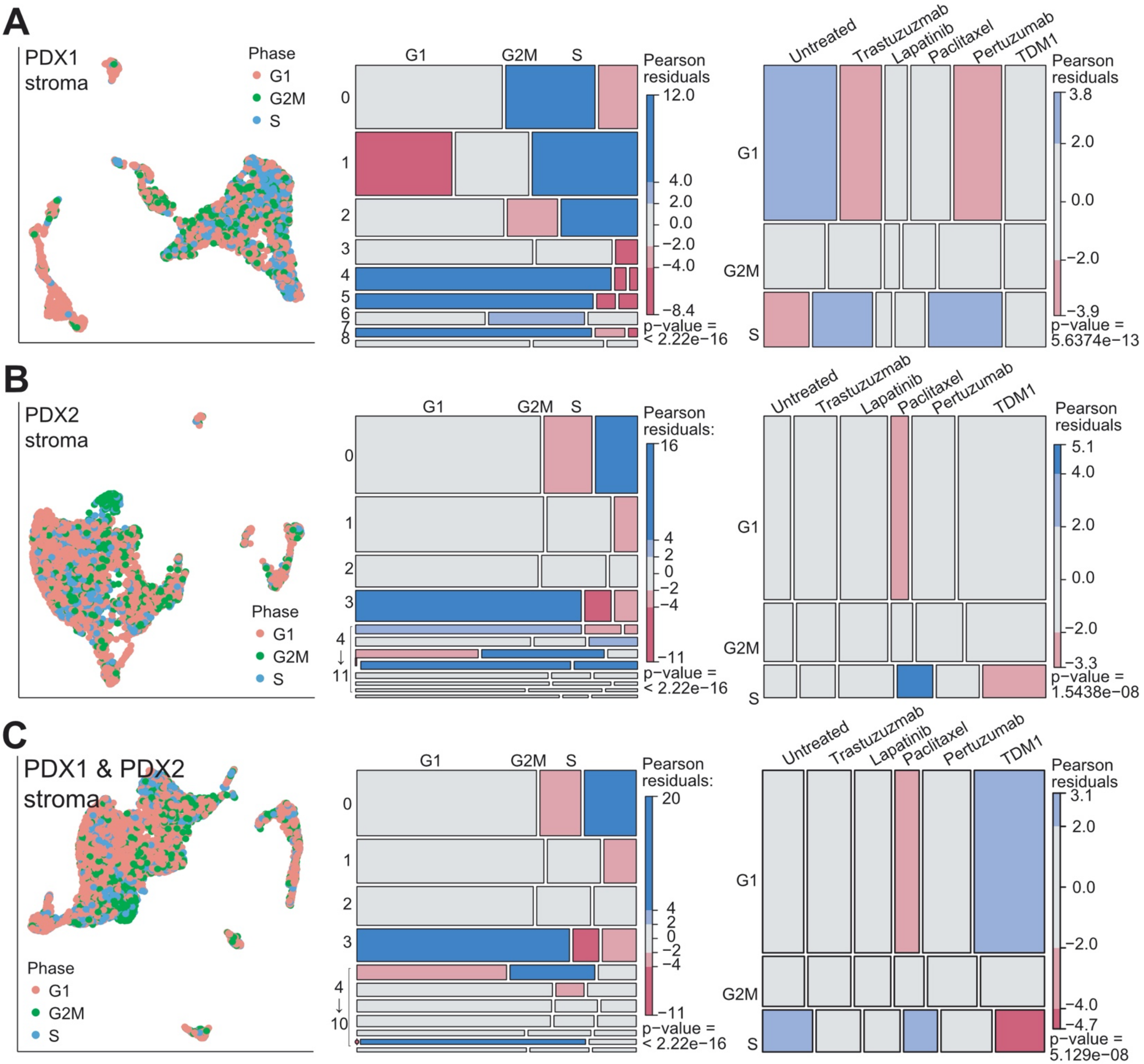

Supplemental Figure 14. Cell cycle gene expression analysis of PDX1 and PDX2 stromal cells. (**A-C**), UMAP clustering of PDX1 stroma (**A**), PDX2 stroma (**B**) or stroma from both PDXs combined (**c**). Stroma cells are colored according to the cell cycle phase (G1, G2M or S phase). Middle and right plots - cell distribution among clusters depending on cell cycle phase. Size of each box is proportional to number of cells within a cluster from given cell cycle phase (middle panel) or treatment (right panel). Red color indicates lower than expected frequency, blue – higher than expected. P value of χ^2 test is shown.

SUPPLEMENTAL METHODS

STAR-FISH data analysis

Hierarchical clustering: For patient i, where i= $1, 2, .., 16$ for the TDM-1 cohort and $i = 1, 2, \ldots 17$ for the Norwegian cohort, denote the number of pretreatment samples as n_{i0} and the number of posttreatment samples as n_{i1}. For each sample j, where $j = 1, 2, \ldots, n_{it}$, we obtained the number of cells assigned to each genotype: m_{itja} represents the number of wildtype (WT) cells, m_{itjb} represents the number of cells with the *PI3KCA* H1047R mutation and no *HER2* amplification (Mut), m_{itjc} represents the number of cells with the *PIK3CA* H1047R mutation and *HER2* amplification (Mut_Amp), m_{itjd} represents the number of cells with no *PIK3CA* H1047R or wildtype signal and with HER2 amplification (Amp), and m_{itje} represents the number of cells with *PIK3CA* wildtype signal and with HER2 amplification (WT_Amp). Thus, each sample has a total number of $m_{itj} = m_{itja} + m_{itjb} + m_{itjc} + m_{itjd} + m_{itje}$ cells. Each patient has a total number of $m_{itk} = \sum_{j=1,2,\ldots,n_{it}} m_{itjk}$ cells per genotype and a total number of $m_{it} = \sum_{j=1,2,\ldots,n_{it}} m_{itj} = \sum_{k\in\{a,b,c,d,e\}} m_{itk}$ cells at timepoint t. Finally, the frequency of genotype k for patient i at timepoint t is $p_{itk} = m_{itk}/m_{it}$. We used the patient genotype frequencies to perform hierarchical clustering of patients separately for each cohort and timepoint. Hierarchical clustering was done using complete linkage clustering.

Diversity index calculation: For each patient i and sample j, we calculated the frequency of each cell type k, $p_{itjk} = m_{itjk}/m_{itj}$, for $k \in \{a, b, c, d, e\}$. We then calculated the Shannon index for each sample: $H_{itj} = -\sum_{k\in\{a,b,c,d,e\}} p_{itjk} ln\,(p_{itjk})$. We also calculated the overall Shannon index for each patient pre- and post-treatment (when data was available post-treatment) as $H_{it} = -\sum_{k\in\{a,b,c,d,e\}} p_{itk} ln\,(p_{itk})$.

Likelihood of heterogeneity calculation: For each patient i at each timepoint $t = 0, 1$, we used a likelihood ratio test to investigate whether it is more likely that all of patient $i's$ samples at timepoint t originated from the same cell population (null hypothesis), or each patient sample at time t has its own unique cell population (alternative hypothesis). Under the assumption that the cell population is multinomially distributed, the null log likelihood for patient i at time t is

$\ell_{it}^{0}(p_{it}) \sim \sum_{k\in\{a,b,c,d,e\}} m_{itk} \ln\ (p_{itk})$ and the alternative log likelihood is $\ell_{it}^{1}(p_{it}) \sim \sum_{j=1,2,\dots,n_{it}} \sum_{k\in\{a,b,c,d,e\}} m_{itjk} \ln(p_{itjk})$. The resulting likelihood ratio test statistic is $\lambda_{it} = -2[\ell_{it}^{0}(p_{it}) - \ell_{it}^{1}(p_{it})]$, where λ_{it} is chi-squared distributed with $4\ (n_{it} - 1)$ degrees of freedom. This results in a separate p-value for each patient and timepoint.

Spatial analysis: For each sample, we obtained the 1024x1024 pixel square image containing the spatial distribution of measured cells. We then performed *k*-means clustering with *k*=2,3,4,5 separately for each sample and mutation. We calculated the ratio of between-cluster sum of squares versus the total sum of squares, which represents how closely clustered cells with a given mutation are. A higher ratio suggests cells are clustered tightly together whereas a lower ratio suggests that cells are randomly distributed throughout the sample We then used the Wilcoxon rank-sum test to test whether there is a significant difference in the spatial distribution between pre- and post-treatment samples in the Norwegian Cohort and responders and non-responders using the pre-treatment samples in both cohorts.

Genotype topology: Using the 1024x1024 pixel square images described above, 2-dimensional kernel density estimation was used to create contour plots for each sample and mutation. Contour lines that are closer together suggest cells with the corresponding mutation are tightly clustered together. If there were not enough cells to estimate the 2D density for a given cell type, no contours corresponding to that genotype are drawn on the topology map.

Analysis of changes in diversity: We used a bootstrap approach to infer whether tumor diversity within each patient changed pre- vs post-treatment. For sample *j* of patient *i* at time *t*, there are m_{itj} cells. We then sampled a new set of m_{itj} cells with replacement from those m_{itj} cells and calculated the overall Shannon index, H_{it}, and the likelihood of heterogeneity test statistic, λ_{it}. We repeated this process 1,000 times to generate 1,000 bootstrapped diversity metrics. The 2.5th and 97.5th quantiles were used to generate 95% confidence intervals. The change in diversity pre- and post-treatment was considered significant if the pre- and post-treatment confidence intervals did not overlap. The direction of change

was determined by the sign of $H_{i2} - H_{i1}$ and $\lambda_{i2} - \lambda_{i1}$ for the Shannon index and likelihood score, respectively.

CycIF details

Fluorescence Microscopy: Fluorescently stained slides were scanned on the Zeiss AxioScan.Z1 (Zeiss, Germany) with a Colibri 7 light source (Zeiss). The filter cubes used for image collection were DAPI (Zeiss 96 HE), Alexa Fluor 488 (AF488, Zeiss 38 HE), AF555 (Zeiss 43 HE), AF647 (Zeiss 50) and AF750 (Chroma 49007 ET Cy7). The exposure time was determined individually for each slide and stain to ensure good dynamic range but not saturation. Full tissue scans were taken with the 20x objective (Plan-Apochromat 0.8NA WD=0.55, Zeiss) and stitching was performed in Zen Blue image acquisition software (Zeiss).

Quenching Fluorescence Signal: After successful scanning, slides were soaked in 1x PBS for 10 – 30 minutes in a glass Coplin jar, waiting until glass coverslip slid off without agitation. Quenching solution containing 20 mM sodium hydroxide (NaOH) and 3% hydrogen peroxide (H2O2) in 1 x PBS was freshly prepared from stock solutions of 5 M NaOH and 30% H2O2, and each slide placed in 10 ml quenching solution. Slides were quenched under incandescent light, for 30 minutes for FFPE tissue slides. Slides were then removed from chamber with forceps and washed 3 x 2 min in 1 x PBS. The next round of primary antibodies was applied, diluted in blocking buffer as previously described, and imaging and quenching were repeated over ten rounds for FFPE tissue slides.

Cyclic IF quantification and analysis: Each image acquired during the Cyclic IF assay was registered based on DAPI features acquired from each round of staining (1). Cellpose, a generalist algorithm for cellular segmentation (2), was used to generate nuclear and cell masks by classifying pixels on the basis of a DAPI or E-cadherin antibody staining, respectively. The following parameters were used for Cellpose segmentation: cells -diameter=30 pixels, flow_threshold=0.6, min_size=113; nuclei -diameter=30, flow_threshold=0, min_size=28. Nuclei with no E-cadherin staining (non-epithelial cells) were expanded by 5 pixels to approximate the cytoplasm. Subcellular regions (e.g., cytoplasm, membrane) were derived from nuclei and cell segmentation results and mean intensity of each

subcellular region was extracted using in-house software [https://gitlab.com/engje/cmif]. Mean intensity used for downstream analysis was selected for each marker based on its biologically-relevant subcellular region (e.g., CK19 - cytoplasm, Ki67 - nuclei). Since the cell membrane is difficult to segment accurately, for membrane markers such as HER2, the brightest 25% of pixels in the segmented region were used for analysis. The last round DAPI image was used to filter out cells lost during each round of Cyclic IF staining.

SUPPLEMENTAL REFERENCES

1. Young Hwan C, Thibault G, Madin O, Azimi V, Meyers C, Johnson B, et al. Deep learning based Nucleus Classification in pancreas histological images. *Annu Int Conf IEEE Eng Med Biol Soc.* 2017;2017:672-5.
2. Stringer C, Wang T, Michaelos M, and Pachitariu M. Cellpose: a generalist algorithm for cellular segmentation. *Nat Methods.* 2021;18(1):100-6.

Image J macro for STAR-FISH signal count

```
v = getInfo("image.filename");
l = lengthOf(v);
v1 = substring(v, 0, l-4);
v2 = substring(v, 0, l-5);
p = getDirectory("image");
run("Duplicate...", v);
selectWindow(v1+"-1.tif");
run("8-bit");
setAutoThreshold("Default dark");
waitForUser("Adjust brightness and threshold. Click OK when ready");
run("Convert to Mask");
run("Fill Holes");
run("Watershed");
run("Analyze Particles...", "size=200-Infinity pixel circularity=0.00-1.00 show=Outlines clear include add");
selectWindow("Drawing of "+v1+"-1.tif");
save(p+"Drawing of "+v1+".tif");
close();
roiManager("Show All with labels");


selectWindow(v2+"2.tif"); //needs tresholding
run("8-bit");
waitForUser("Adjust brightness and threshold. Click OK when ready");
run("Clear Results");
//run("Invert");
```

```
n = roiManager("count");
for (i=0; i<n; i++) {
   roiManager("select", i);
run("Analyze Particles...", "size=2-200 pixel circularity=0.00-1.00 show=Nothing display");
roiManager("deselect");
print(i+1, ",", nResults);
run("Clear Results");
}

selectWindow("Log");
save(p+"Log_"+v2+"2.txt");
saveAs("Text", "/Users/…/Log_"+v2+"2.txt");

print("\\Clear");

selectWindow(v2+"0.tif"); //needs tresholding
run("8-bit");
waitForUser("Adjust brightness and threshold. Click OK when ready");
run("Clear Results");
//run("Invert");
n = roiManager("count");
for (i=0; i<n; i++) {
   roiManager("select", i);
run("Analyze Particles...", "size=2-20 pixel circularity=0.00-1.00 show=Nothing display");
roiManager("deselect");
print(i+1, ",", nResults);
run("Clear Results");
}

selectWindow("Log");
save(p+"Log_"+v2+"0.txt");
saveAs("Text", "/Users/…/Log_"+v2+"0.txt");

print("\\Clear");

selectWindow(v2+"3.tif"); //needs tresholding
run("8-bit");
waitForUser("Adjust brightness and threshold. Click OK when ready");
run("Clear Results");
//run("Invert");
n = roiManager("count");
for (i=0; i<n; i++) {
  roiManager("select", i);
run("Analyze Particles...", "size=1-200 pixel circularity=0.00-1.00 show=Nothing display");
roiManager("deselect")
print(i+1, ",", nResults);
run("Clear Results");
}
```

```
selectWindow("Log");
save(p+"Log_"+v2+"3.txt");
saveAs("Text", "/Users/…/Log_"+v2+"3.txt");

print("\\Clear");

selectWindow(v2+"1.tif"); //needs tresholding
run("8-bit");
waitForUser("Adjust brightness and threshold. Click OK when ready");
run("Clear Results");
//run("Invert");
n = roiManager("count");
for (i=0; i<n; i++) {
  roiManager("select", i);
run("Analyze Particles...", "size=2-200 pixel circularity=0.00-1.00 show=Nothing display");
roiManager("deselect");
print(i+1, ",", nResults);
run("Clear Results");
}

selectWindow("Log");
save(p+"Log_"+v2+"1.txt");
saveAs("Text", "/Users/…/Log_"+v2+"1.txt");


print("\\Clear");

roiManager("save", p+"Roi_"+v1+".zip");

selectWindow(v1+"-1.tif");
run("Set Measurements...", "area centroid limit display redirect=None decimal=0");
n = roiManager("count");
for(i=0; i<n; i++) {
        roiManager("select", i);
        run("Measure");
        x = getResult('X');
        y = getResult('Y');
getVoxelSize(width, height, depth, unit);
x1 = x/width;
y1 = y/height;
        print(i+1, ",", d2s(x1,0), ",", d2s(y1,0));
run("Clear Results");
}

selectWindow("Log");
save(p+"Log_"+v1+"_xy.txt");
saveAs("Text", "/Users/…/Log_"+v1+"_xy.txt");
```
