# Supplementary material for: The impact of tumor epithelial and microenvironmental heterogeneity on treatment responses in HER2+ breast cancer
Source: JCI Insight. 2021 Jun 8;6(11):e147617. doi: 10.1172/jci.insight.147617 (PMC8262355; doi:10.1172/jci.insight.147617)
Supplement: Supplemental Table 3 [file jciinsight-6-147617-s071.pdf]

Supplemental Table 3. Cyclic immunofluorescence (CyCIF) gating strategy. Combinations of markers related to specific cell types were used to assign individual cells to a particular cell type.

CELL TYPE	GATING
**Tumor**	**CD31-, any cytokeratin+**
Luminal tumor	CK19, CK7 or CK8 +
Basal Tumor	CK5 or CK14 +
Mesenchymal Tumor	Vimentin+, cytokeratin +
**Immune**	**CD31- and cytokeratin-; CD45, CD68, CD3 or CD4+**
Macrophage	CD68+, CD31-, cytokeratin- (+/- GRNZB and PD1)
B cell	CD20+, CD45+, CD68-, CD31-, cytokeratin-
CD8 T cell	CD8+, CD45 or CD3+, CD20-, CD68-, CD31-, cytokeratin- (+/- GRNZB, PD1 and FoxP3)
CD4 T cell	CD4+, CD8-, CD20-, CD68-, CD31-, cytokeratin- (+/- GRNZB, PD1 and FoxP3)
**Endothelial**	**CD31+**
**Stromal**	**CD31-, cytokeratin-, CD45-, CD3-, CD4-, CD68-**
Stromal cell types	Defined above, (+/- αSMA, Vimentin, Podoplanin)
Proliferation	Ki67+
