# Supplementary material for: The impact of tumor epithelial and microenvironmental heterogeneity on treatment responses in HER2+ breast cancer
Source: JCI Insight. 2021 Jun 8;6(11):e147617. doi: 10.1172/jci.insight.147617 (PMC8262355; doi:10.1172/jci.insight.147617)
Supplement: Supplemental Table 12 [file jciinsight-6-147617-s069.pdf]

Supplemental Table 12. Antibodies used for immunostaining.

Antibody	Host species	Company	Cat. number	Dilution used	Antigen retrieval	Use
Cleaved caspase-3	rabbit	Cell Signaling	9661	1:100	pH6	IHC/IF
Phospho-histone H3	rabbit	Abcam	ab5176	2.5ug/ml	pH6	IHC/IF
HER2	mouse IgG1	Abcam	ab16901	5ug/ml	pH6	IHC/IF
Phospho-EGFR	rabbit	Abcam	ab40815	4.3ug/ml	pH6	IHC/IF
Estrogen receptor	rabbit	ThermoFisher	RM9101S1	1:1000	pH6	IHC/IF
Anti-rabbit-AlexaFluor488	goat	ThermoFisher	A11034	20ug/ml		IHC/IF
Anti-mouse IgG1-AlexaFluor594	goat	ThermoFisher	A21125	20ug/ml		IHC/IF
αSMA	mouse	Santa Cruz	sc-32251	1:200	pH6/pH9	CyCIF
CD20	rabbit	abcam	ab198941	1:50	pH6/pH9	CyCIF
CD3	rabbit	abcam	ab213608	1:50	pH6/pH9	CyCIF
CD31	rabbit	abcam	ab218582	1:100	pH6/pH9	CyCIF
CD4	rabbit	abcam	ab196147	1:100	pH6/pH9	CyCIF
CD45	rabbit	abcam	ab214437	1:50	pH6/pH9	CyCIF
CD68	mouse	Biolegend	916104	1:50	pH6/pH9	CyCIF
CD8	mouse	abcam	ab213017	1:50	pH6/pH9	CyCIF
CK14	mouse	abcam	ab212547	1:400	pH6/pH9	CyCIF
CK19	mouse	Biolegend	628502	1:200	pH6/pH9	CyCIF
CK5	rabbit	Biolegend	905501	1:200	pH6/pH9	CyCIF
CK7	rabbit	abcam	ab185048	1:400	pH6/pH9	CyCIF
CK8	rabbit	abcam	ab192467	1:200	pH6/pH9	CyCIF
Ecad	rabbit	abcam	ab201499	1:100	pH6/pH9	CyCIF
ER	rabbit	abcam	ab205851	1:100	pH6/pH9	CyCIF
FoxP3	mouse	Biolegend	320102	1:50	pH6/pH9	CyCIF
GRNZB	rabbit	abcam	ab219803	1:100	pH6/pH9	CyCIF
HER2	mouse	Santa Cruz	sc-33684	1:50	pH6/pH9	CyCIF
Ki67	rabbit	Cell Signaling	12075	1:400	pH6/pH9	CyCIF
PD1	rabbit	abcam	ab201825	1:50	pH6/pH9	CyCIF
PDPN	mouse	Biolegend	916606	1:50	pH6/pH9	CyCIF
Vimentin	rabbit	Cell Signaling	9854	1:400	pH6/pH9	CyCIF
